# Traumatic brain injury and post-acute decline: what role does environmental enrichment play? A scoping review

**DOI:** 10.3389/fnhum.2013.00031

**Published:** 2013-04-17

**Authors:** Diana Frasca, Jennifer Tomaszczyk, Bradford J. McFadyen, Robin E. Green

**Affiliations:** ^1^Graduate Department of Rehabilitation Science, University of TorontoToronto, ON, Canada; ^2^Cognitive Neurorehabilitation Sciences Laboratory, Toronto Rehabilitation InstituteToronto, ON, Canada; ^3^Département de Réadaptation, Université LavalQuébec City, QC, Canada; ^4^Centre Interdisciplinaire Recherche en Réadaptation et Intégration SocialeQuébec City, QC, Canada

**Keywords:** traumatic brain injury, environmental enrichment, post-acute decline, adult, moderate to severe, post-discharge, transition home

## Abstract

**Objectives:** While a growing number of studies provide evidence of neural and cognitive decline in traumatic brain injury (TBI) survivors during the post-acute stages of injury, there is limited research as of yet on environmental factors that may influence this decline. The purposes of this paper, therefore, are to (1) examine evidence that environmental enrichment (EE) can influence long-term outcome following TBI, and (2) examine the nature of post-acute environments, whether they vary in degree of EE, and what impact these variations have on outcomes.

**Methods:** We conducted a scoping review to identify studies on EE in animals and humans, and post-discharge experiences that relate to barriers to recovery.

**Results:** One hundred and twenty-three articles that met inclusion criteria demonstrated the benefits of EE on brain and behavior in healthy and brain-injured animals and humans. Nineteen papers on post-discharge experiences revealed that variables such as insurance coverage, financial, and social support, home therapy, and transition from hospital to home, can have an impact on clinical outcomes.

**Conclusion:** There is evidence to suggest that lack of EE, whether from lack of resources or limited ability to engage in such environments, may play a role in post-acute cognitive and neural decline. Maximizing EE in the post-acute stages of TBI may improve long-term outcomes for the individual, their family and society.

## Introduction

Moderate to severe traumatic brain injury (TBI) is a ubiquitous injury: studies suggest an annual incidence of upwards of 20–60 per 100,000 (Narayan et al., 2002; Bruns and Hauser, 2003; Cassidy et al., 2004; C.I.H.I., 2006). Many of these injuries are sustained in young adulthood (C.I.H.I., 2006; Faul et al., 2010) and result in significant impairment to cognitive, motor, and emotional functioning. Predominant and persisting deficits to executive functioning, attention, memory, and speed of processing compromise psychosocial functioning and quality of life (Sander et al., 2001; Hawthorne et al., 2009; Resch et al., 2009). Because these deficits prevent many TBI survivors from returning to pre-injury levels of activity and participation (Dikmen et al., 1983, 1995; Lezak, 2004; Christensen et al., 2008), successful community integration is now recognized as a primary goal of rehabilitation for persons with brain injury (Sander et al., 2010).

The consequences of brain injury are particularly concerning given the high incidence of TBI. Murray and Lopez (1997) predicted that by 2020, TBI will be the third leading cause of disability in the world. Considering that males aged 15–24 years have the highest incidence of TBI (Pickett et al., 2004; C.I.H.I., 2006; Faul et al., 2010), this can mean decades of disability and lost productivity. Not surprisingly, the annual burden of acute care and rehabilitation in North America is estimated to be in the billions of dollars (SMARTRISK, 2006; Faul et al., 2010).

A theoretically intriguing and clinically important question that is emerging from the literature is whether an impediment to recovery and a contributing factor to failed community integration after moderate to severe TBI is cognitive and brain deterioration in the post-acute stages after brain injury. TBI recovery studies typically show an asymptotic pattern of recovery, with rapid improvement within the first weeks and months of injury, followed by a slower rate of improvement and then a plateau with limited measureable recovery thereafter (Basso, 1989; Heinemann et al., 1995; Blatter et al., 1997; Holbrook et al., 1999; Christodoulou et al., 2001; Farne et al., 2004). However, not only do many fail to return to pre-injury levels of function when they reach that plateau (Christensen et al., 2008), but there is growing evidence that a subset of TBI survivors show cognitive deterioration.

A number of studies that have examined post-acute cognitive changes in TBI survivors have demonstrated that across domains of functioning, a combination of maintenance, further recovery and frank declines are observed (Ruff et al., 1991; Millis et al., 2001; Sander et al., 2001; Himanen et al., 2006; Salmond et al., 2006; Till et al., 2008). For example, Till et al. (2008) showed that nearly 30% of their sample of moderate-severe TBI patients showed clinically significant decline (as measured by the reliable change index) in two or more domains of cognitive functioning.

In examining changes of the brain during the post-acute stages of TBI, imaging studies have also shown evidence of deterioration, including decreased cerebral blood flow (Kim et al., 2010), declines in whole brain volume (Blatter et al., 1997; Mackenzie et al., 2002; Trivedi et al., 2007; Ng et al., 2008; Sidaros et al., 2009; Hudak et al., 2011), atrophy of discrete gray, and white matter structures including the hippocampus and corpus callosum (Wilson et al., 1988; Bigler et al., 1997; Levine et al., 2008; Ng et al., 2008; Sidaros et al., 2008), lesion expansion (Ng et al., 2008); and reduced white matter integrity as measured by diffusion tensor imaging (Bendlin et al., 2008; Greenberg et al., 2008; Sidaros et al., 2008; Warner et al., 2010; Farbota et al., 2012).

Correlations between brain and behavioral decline have also been observed. In one study, 24 TBI survivors underwent MRI scans at 8 weeks and 12 months post-injury. The authors not only demonstrated increased atrophy during this time period, but also negatively correlated outcomes on the Glasgow Outcome Scale (Sidaros et al., 2009). In other studies, Hudak et al. (2011) found that decreases in brain volume correlated with depressive symptoms in the post-acute phase, and Farbota et al. (2012) demonstrated that diffusion tensor imaging findings (fractional anisotropy values) and neuropsychological task performance were positively correlated.

Given mounting evidence revealing post-acute decline, we suggest that it is important at this stage of research to begin to consider what factors may hold the potential to influence, and in particular, offset this decline.

One factor that may play a role is “environmental enrichment” (EE). As we will discuss further in the next section, EE broadly refers to enhanced stimulation, associated with (1) environments that provide access to cognitive as well as physical and social stimulation, and (2) conditions that encourage maximal participation. An extensive body of literature shows positive correlations between EE and cognitive and neuronal status. (Scarmeas and Stern, 2003; Will et al., 2004; Simpson and Kelly, 2011). There have also been some findings to suggest that EE influences post-acute decline. In Till et al.'s (2008) study of post-injury cognitive decline, the authors found a relationship between hours of therapy at 5 months post-injury and degree of cognitive decline from 12 to 24 months. They concluded that lack of access to complex and enriched environments, due in part to limited access to resources, may play a critical a role in decline. In another study, Miller and Green (in press) found that greater hippocampal volume loss in the chronic stages of TBI (12–24 months post-injury) was negatively correlated with degree of cognitive stimulation reported at 5 months post-injury.

These few but important findings raise the question whether post-acute decline in these survivors was influenced by the extent of enrichment, or lack thereof, in the environments to which they were discharged following the early and intensive months of therapy. A reduction in the level of enrichment in the later stages of recovery from TBI might occur when the number and hours of therapies are reduced or when patients move from in-patient neurorehabilitation back to the home environment. Additionally, TBI survivors may return to environments that are indeed complex and enriched, but without the expertise of therapists actively providing supports and adaptations to the environments, patients may be unable to engage due to cognitive, emotional and/or physical impairments that render the environments overwhelming or inaccessible.

The purposes of this paper, therefore, are to (1) examine evidence that EE can influence long-term outcome following TBI, and (2) examine the nature of post-acute environments, whether they vary in degree of EE, and what impact these variations have on outcomes. To accomplish these aims, we will undertake a scoping review summarizing literature related to EE and post-acute environments.

## Scoping review methods

This paper addresses two of the reasons for undertaking a scoping review identified by Arksey and O'Malley (2005): to summarize and disseminate research findings and to identify research gaps in the existing literature. The scoping review typically unfolds in five steps: (1) identify the research question; (2) identify all pertinent studies; (3) select the studies for detailed analysis; (4) chart the data according to key concepts; and (5) collate and summarize the findings of the selected studies (Arksey and O'Malley, 2005; Rumrill et al., 2010):

### Identify the research question

The research questions addressed in this paper are whether (1) EE can influence long-term recovery, and (2) post-discharge environments vary in degree of EE, and whether such variations influence outcomes.

### Identify all pertinent studies

The literature review aimed to identify a comprehensive set of articles detailing the effects of EE in animals and humans, and the post-discharge experiences related to recovery and regaining independence in the post-acute stages after TBI. Articles that addressed these topics were obtained through use of a traditional keyword-driven electronic search guided by the following terms: TBI; recovery; EE; environmental complexity; active lifestyle; stimulation; neuroplasticity; cognitive reserve; intervention; multi-disciplinary; multi-contextual; post-rehabilitation; transition home; barriers; community integration; re-engagement. Peer-reviewed journals were searched using the PubMed and Cochrane Collaboration research databases as well as the Google Scholar search engine, for articles published between 1987 and 2012. Additionally, hand searches were conducted of references from key articles to follow up on seminal work and promising literature that might not have been captured by the databases used.

### Select studies for detailed analysis

To be considered for inclusion in the review, articles had to meet the following criteria: (1) describe EE (or components of EE) in animals or adult humans or (2) describe the post-discharge experience, and more specifically, the transition from hospital to home in adult brain-injury survivors; and (3) be available in English. No methodological limitations were applied to screen for levels of evidence.

### Chart data according to key concepts

Articles that met the inclusion criteria were reviewed in detail and categorized based on population examined, methodology, and study objectives to discover commonalities and provide connections between the sets of literatures reviewed.

### Collate and summarize findings

The results are presented to correspond with the objectives of this paper. A numerical summary of the articles included is followed by a summary of the literature.

## Results

Database searches identified 2053 articles. Of these, 142 were included in the review based on the selection criteria listed above. Table 1 provides a brief summary of the articles that were included. Tables 2 and 3 provide a more detailed summary of the papers that specifically addressed EE in brain-injured animals and humans, as well as EE and post-discharge experiences, respectively. Tables 2 and 3 detail the populations, methods, objectives, and main findings of the articles that were included.

**Table 1:** **Numerical summary of articles reviewed**.

**Literature topic**	**Number of studies**	**Methodologies**	**Populations**
Beneficial effects of EE	123: animals—55; humans—68	*Animal literature*: quantitative, experimental; *Human literature:* quantitative, correlational, observational, intervention	Healthy and brain-injured animals, humans
Post-discharge experiences	19	Qualitative, observational, correlational, reviews, case study	Brain-injured humans

**Table 2:** **Detailed summary of articles included in “Brain-injured animals and EE” and “Brain-injured humans and EE” scoping review**.

**Authors**	**Methods**	**(1) Main objectives and (2) Findings**
**BRAIN-INJURED ANIMALS AND EE**		
Hamm et al., 1996	Brain-injured and sham-injured rats in EE and standard environment (SE)	(1) Determine whether exposure to EE would promote recovery of cognitive function; (2) Brain-injured rats in EE vs. SE: EE rats showed more improvement in Morris Water Maze task; Brain-injured rats in EE vs. sham-injured: performed at same level.
Johansson and Ohlsson, 1996	Brain-injured rats randomly assigned to EE, social-stimulation only or physical-stimulation only environment	(1) Determine relative importance of social and physical activity to EE; (2) No difference in infarct size between groups. EE group performed better than physical group in all tests, better than social group on rotating pole. With time EE group performed better than social group in limb placement, climbing, inclined plane. Social group performed better than physical group on inclined plane and in climbing at all times, by 13 weeks also in limb placement test and on beam.
Passineau et al., 2001	Brain-injured and sham-injured rats randomly assigned to EE and SE	(1) Examine effect of EE on behavior and on histological integrity of brain tissue selectively vulnerable to brain trauma; (2) Injured animals in EE showed shorter latencies to find platform in Morris Water Maze task vs. injured/SE animals on day 12 post-TBI. Both injured groups showed deficits vs. sham groups. At 14 days post-TBI, EE animals had approximately 2× smaller lesion areas in regions of cerebral cortex posterior to injury epicenter compared to injured/SE animals. Overall lesion volume for entire injured cortical hemisphere was smaller in animals recovering in EE.
Dobrossy and Dunnett, 2001	Brain-injured rodents; review	(1) Review degree to which housing conditions or behavioral training can modify survival, integration or function of transplanted tissue; (2) Behavioral training experience can promote behavioral, and functional compensation, and influence neuroplasticity at cellular, and systems levels of neuronal reorganization.
Johansson, 2003	Brain-injured rats; review	(1) Review influence of post-ischemic environmental factors, possible clinical implications; (2) EE improves functional outcome, increases dendrite branching, number of dendritic spines in contralateral cortex, influences expression of many genes, modifies lesion-induced stem cell differentiation in hippocampus.
Dobrossy and Dunnett, 2004	Brain-injured rats with and without neural grafts randomly assigned to EE and SE	(1) Examine effects of differential housing conditions on striatal graft morphology and functional recovery; (2) Functional recovery accompanied by reduction in infarct size and more afferent connections.
Will et al., 2004	Brain-injured rats; review	(1) Compare three main non-invasive therapeutic strategies for achieving rehabilitation after brain damage: EE, physical exercise, specific formal training; (2) EE increased neurogenesis in hippocampus and up-regulation of neurotropic factors (e.g., NGF) that result in decreased spontaneous apoptosis and increased neuronal survival.
Gobbo and O'Mara, 2004	Brain-injured rats housed under EE or SE, 6 weeks before, 4 weeks after surgery	(1) Investigate if EE can protect rats against the cognitive and neurological consequences of transient ischemia; (2) EE improved learning and memory; does not protect against actual loss of CA1 pyramidal cells. Brain-derived neurotrophic factor levels were increased.
Lippert-Gruener et al., 2007	Brain-injured and sham-injured rats assigned to EE, EE + multi-modal early onset stimulation (MEOS), or SE	(1) Investigate effects of EE, EE+ MEOS, and SE on cognitive and motor function, and cortical lesion volume; (2) Rats in EE and EE+MEOS demonstrated improvement over SE, but no change in lesion size.
Pereira et al., 2007	Brain-injured and sham-injured rats randomly assigned to EE and SE	(1) Examine effects of daily EE on memory deficits in water maze and cerebral damage; (2) Spatial reference, working memory impairments were completely reversed by EE; Reduction of both hippocampal volume and cortical area, ipsilateral to arterial occlusion, no EE effect on morphological measurements.
Hoffman et al., 2008	Brain-injured and sham-injured rats randomly assigned to early EE, delayed EE, continuous EE or no EE	(1) Examine whether EE-mediated benefits are dependent on exposure to EE during neurobehavioral training; (2) A3 cell loss significantly attenuated in TBI + continuous EE group vs. TBI + no EE group. Beam-walking was facilitated in TBI groups that received early or continuous EE vs. those receiving delayed or no EE. Cognitive training enhanced in TBI groups that received continuous or delayed EE vs. early or no EE groups.
Sozda et al., 2010	Brain-injured and sham-injured rats assigned to typical EE, EE –social, EE –stimuli, SE, SE +stimuli	(1) Investigate effects of typical EE, EE –social, EE –stimuli, SE, SE +stimuli on motor and cognitive function, lesion volume, brain volume loss; (2) Typical EE groups performed same as sham group, and showed most improvement compared to other TBI groups in terms of spatial learning and memory retention, lesion size reduction.
Sun et al., 2010	Brain-injured rats randomly assigned to EE or SE	(1) Investigate effects of EE on cognitive impairment, levels of BDNF and NMDA receptor subunit 1 (NR1) and subunit 2B (NR2B) in hippocampus; (2) EE exposure improved spatial cognitive performance and non-spatial memory performance. EE increased levels of BDNF and NR1 protein in hippocampus.
Matter et al., 2010	Brain-injured or sham-injured rats randomly assigned to 8 groups receiving continuous, early or delayed EE with either 1 or 2 weeks of exposure	(1) Further assess effects of time of initiation and duration of EE on neurobehavioral recovery by evaluating and directly comparing all the temporal permutations; (2) Motor ability was enhanced in TBI groups that received early EE (i.e., during testing) vs. standard housing. Acquisition of spatial learning facilitated in groups receiving delayed EE (i.e., during training).
De Witt et al., 2011	Brain-injured and sham-injured rats randomly assigned to EE, EE (2 h), EE (4 h), EE (6 h), or SE	(1) Determine whether abbreviated EE (i.e., rehab-relevant dose response) confers benefits similar to typical EE; (2) TBI + EE (2 h) and TBI + EE (4 h) groups not different from TBI + STD group in behavioral assessment. TBI + EE (6 h) group exhibited enhancement of motor and cognitive performance when compared with TBI + STD group, TBI + EE (2 h) and TBI + EE (4 h) groups, and did not differ from TBI + EE (typical) group.
Cheng et al., 2012	Brain-injured and sham-injured rats randomly assigned to 3 weeks of EE or SE. In phase 2: half of rats in EE transferred to SE conditions (TBI + EE + SE and sham + EE + SE; re-assessed 1/month for 6 months)	(1) Determine whether EE-mediated motor and cognitive benefits persist after its withdrawal; (2) TBI + EE and TBI + EE + STD groups performed better in the water maze than the TBI + STD group, did not differ from one another. Data replicate several studies showing that EE enhances recovery after brain injury, and extend by demonstrating that cognitive benefits are maintained for at least 6 months post-rehabilitation.
Shin et al., 2013	Brain-injured and sham-injured rats assigned to EE or SE	(1) Investigate effects of EE on substantia nigra gene expression; (2) EE-induced gene alterations after TBI included genes important for signal transduction, in particular calcium signaling pathways, membrane homeostasis, and metabolism.
Monaco et al., 2013	Brain-injured and sham-injured rats assigned to EE or SE	(1) Assess effect of EE on functional and histological outcome in female rats after TBI; (2) EE improved motor function and spatial learning; reduced lesion size and increased hippocampal cell survival.
**BRAIN-INJURED HUMANS AND EE**		
Blackerby, 1990	Acute moderate-severe TBI (*n* = 145); retrospective; quantitative	(1) Investigate effects of different levels of rehabilitation intensity on length of stay in two hospital-based coma and acute rehabilitation populations; (2) After increasing treatment intensity and changes in case management, patients were discharged an average of 1.5 months earlier than before changes.
Toglia, 1991	Brain injury; concept paper	(1) Review literature on learning and generalization and direct applications to treatment; (2) Five components identified in cognitive psychology literature as critical to process of generalization: (a) use of multiple environments, (b) identification of criteria for transfer, (c) meta-cognitive training, (d) emphasis on processing strategies, and (e) use of meaningful activities.
Spivack et al., 1992	Acute moderate-severe TBI (*n* = 95); prospective; quantitative: repeated measures	(1) Examine effects of intensity of treatment and length of stay during inpatient rehabilitation hospitalization; (2) Patients with longer length of stay (LOS) made more progress across all outcome variables than patients with shorter LOS; In long LOS group, two treatment-intensity groups initially equivalent, and at discharge high-intensity treatment group surpassed low-intensity treatment group.
Willer et al., 1999	Post-acute/chronic severe TBI (*n* = 46); prospective; quantitative: case control matched design, repeated measures	(1) Compare outcomes of a post-acute residential rehabilitation program with a matched sample receiving limited services in their homes or on an outpatient basis; (2) Individuals who received intensive rehabilitation services in community-based residential program exhibited considerable improvement in functional abilities (cognitive skills, motor skills). Treatment group showed greater improvement in community integration.
Sohlberg et al., 2000	Chronic moderate-severe TBI, ABI (*n* = 14); prospective; quantitative: repeated measures	(1) Compare attention processing training with an educational and support method; (2) 10 weeks of brain injury education seemed most effective in improving self-reports of psychosocial function. Attention process training influenced self-reports of cognitive function, had stronger influence on performance of executive attention tasks. Vigilance, orienting networks showed little specific improvement.
Cicerone et al., 2000	TBI/Stroke; review	(1) Establish evidence-based recommendations for clinical practice of cognitive rehabilitation from methodical review of scientific literature concerning effectiveness of cognitive rehabilitation; (2) Attention deficits: limited evidence exists for generalization of benefits attributable to attention remediation, tendency to observe gains on tasks most closely related to training tasks; Multi-modal interventions: can significantly improve neuropsychological performance in many skill areas. Maintenance, generalization of benefits from cognitive rehabilitation greatest when treatment is provided for appropriately long periods of time, when efforts are made by clinician and patient to identify and apply interventions to personally relevant areas of functioning, when patients are able to assume responsibility for using compensatory strategies in everyday functioning.
De Weerdt et al., 2000	Acute stroke (*n* = 56); prospective; quantitative: observational	(1) Observe how stroke patients spend their time in a rehabilitation unit; (2) Patients most frequently involved in therapeutic activities, Belgium: 28% of day, Switzerland: 45%. Belgian patients: 27% of day in own room, Swiss: 49% of day. Swiss patients spent nearly 1.5 h per day more in therapy. Differences between two settings could only partially be explained by more favorable patient-staff ratios in Swiss setting.
Fasotti et al., 2000	Post-acute/chronic severe TBI (*n* = 22); prospective; quantitative: repeated measures	(1) Compare the effectiveness of Time Pressure Management (TPM) training with concentration training in which verbal instruction was the key element; (2) TPM produces greater gains than concentration training and appears to generalize to other measures of speed and memory function.
Zhu et al., 2001	Post-acute moderate-severe TBI (*n* = 36); prospective; quantitative: repeated measures	(1) Evaluate effects of different levels of intensive rehabilitation treatment on functional outcome; (2) Increasing amount of rehabilitation from conventional 2–4 h/day improved functional outcome as measured by GOS. More patients in intensive group returned to gainful work, either original or modified job. Improvement most significant in early post-injury period at 2–3 months.
Shiel et al., 2001	Moderate-severe TBI (*n* = 56); prospective; quantitative: repeated measures	(1) Investigate effect of increased intensity of rehabilitation on rate at which independence was regained and duration of hospital admission; (2) Increasing hours per week of therapy can accelerate rate of recovery of personal independence and result in being discharged from hospital sooner. No evidence of ceiling effect of therapeutic intensity beyond which no further response observed.
Park and Ingles, 2001	ABI; meta-analysis	(1) Examine the efficacy of attention rehabilitation; (2) Direct-retraining methods produced only small non-significant improvements in performance. Few studies that attempted to rehabilitate specific skills requiring attention showed statistically significant improvements after training and had considerably larger effect sizes. Results suggest learning that occurs as a function of training is specific, does not tend to generalize or transfer to tasks that differ considerably from those used in training.
Powell et al., 2002	Post-acute/chronic severe TBI (*n* = 94); prospective; quantitative: RCT	(1) Evaluation of multidisciplinary community based outreach rehabilitation; (2) Outreach participants significantly more likely to show gains on Barthel Index, BICRO-39 total score, self-organization, psychological well-being subscales. Strong trends for BICRO personal care and mobility, on FIM+FAM for personal care and cognitive functions.
Slade et al., 2002	Acute stroke/TBI (*n* = 161); prospective; quantitative: RCT	(1) Examined if increased intensity of therapy would decrease length of stay; (2) Accounting for impairment/disability mix, and consequent response of therapy, enhanced levels of physiotherapy and occupational therapy led to benefits for experimental group, resulting in decrease length of stay.
Cifu et al., 2003	Moderate-severe TBI (*n* = 491); prospective; quantitative: RCT	(1) Identify factors relating to intensity of rehabilitation services received and to ascertain relation between injury outcomes, demographics, types of therapy, and intensity of rehabilitation services provided; (2) Findings support assertions that increased therapy intensity, particularly physical and psychological therapies, enhances functional outcomes.
Rath et al., 2003	Chronic mild-severe TBI (*n* = 60); prospective; quantitative: repeated measures	(1) Compare efficacy of a group-treatment protocol using a remedial programme that aims to reduce difficulties in emotional self-regulation, and to facilitate steps used in problem solving with a conventional neuropsychological rehabilitation programme; (2) Participants in innovative group improved in problem solving as assessed using a variety of measures, including (i) executive function, (ii) problem-solving self-appraisal, (iii) self-appraised emotional self-regulation and clear thinking, (iv) objective observer ratings of role-played scenarios. Improvements were maintained at follow-up.
Boman et al., 2004	Mild-moderate TBI (*n* = 10); prospective; experimental: repeated measures	(1) Examine efficacy of cognitive rehabilitation in the patient's home or vocational environment; (2) Positive effect on some measures on impairment level, no differences on activity or participation levels at follow-up; indicates home-based cognitive training improves some attentional and memory functions and facilitates learning of strategies.
Cicerone et al., 2004	Post-acute/chronic moderate-severe TBI (*n* = 56); prospective; quantitative: repeated measures	(1) Evaluate effectiveness of an intensive cognitive rehabilitation program (ICRP) compared with standard neurorehabilitation (SRP); (2) ICRP participants over twice as likely to show clinical benefit on Community Integration Questionnaire. ICRP participants showed improvement in overall neuropsychological functioning; participants with clinically significant improvement on Community Integration Questionnaire showed greater improvement of neuropsychological functioning. Satisfaction with cognitive functioning made significant contribution to post-treatment community integration.
De Wit et al., 2005	Stroke (*n* = 60); prospective; quantitative: observational	(1) Identify differences in use of time by stroke patients in 4 rehabilitation centers in 4 countries; (2) Patients in Belgium and UK spent more time in passive behavior, in rooms, without any interaction compared with patients in Germany and Switzerland. Latter centers had more structured rehabilitation program. May have resulted in more therapy time, more challenging environment for patients, physically and mentally.
Turner-Stokes et al., 2005	Mild-severe ABI; Cochrane review	(1) Assess effects of multi-disciplinary rehabilitation in adults aged 16–65 years; (2) For patients with moderate- severe ABI already in therapy, there was strong evidence that more intensive programmes are associated with earlier functional gains, and “moderate evidence” that continued outpatient therapy could help to sustain gains made in early post-acute rehabilitation.
Cicerone et al., 2005	TBI, stroke; review	(1) Update previous evidence-based recommendations of the Brain Injury Interdisciplinary Special Interest Group of the American Congress of Rehabilitation Medicine for cognitive rehabilitation; (2) Consensus that cognitive rehabilitation should focus on reducing disability, helping restore social role functioning, rather than exclusively on remediation of impairments. Most studies evaluated outcome of interventions at impairment level rather than effect on performance of activities or changes in social participation.
Zhu et al., 2007	Acute/post-acute moderate-severe TBI (*n* = 68); Prospective; RCT	(1) Evaluate the effects of an increase in the intensity of rehabilitation on functional outcome; (2) More patients in the high intensity group than in the control group who achieved a maximum FIM total score at the third month and a maximum Glasgow Outcome Scale score at the second and third months.
Kleim and Jones, 2008	Healthy adult/TBI; review	(1) Review 10 principles of experience-dependent neural plasticity and considerations in applying them to the damaged brain; (2) Optimism that the nature of brain plasticity can be capitalized upon to improve rehabilitation efforts and to optimize functional outcome.
Kennedy et al., 2008	TBI; Systematic review, meta-analysis	(1) Review studies that focused on executive functions of problem solving, planning, organizing and multitasking; (2) Compelling evidence from 10 intervention studies that using step-by-step meta-cognitive strategy instruction improves problem solving, etc. for personally relevant activities or problem situations; Changes more likely to be observed at level of activities and participation in daily living than on standardized tests (i.e., impairment outcomes).
Spikman et al., 2009	ABI (*n* = 75); prospective; quantitative: RCT, repeated measures	(1) Evaluate the effects of a treatment for dysexecutive problems on daily life functioning; (2) Experimental patients resumed previous roles significantly more than before treatment. From post-treatment to follow-up, only experimental group showed further improvement over time; DEX showed decrease of executive complaints similar for both groups. On DEX-therapist, significantly less executive problems after treatment for experimental group. Executive abilities observed by professionals improved more in experimental group.
Toglia et al., 2010	Chronic moderate TBI (*n* = 4); quantitative: single-subject design; repeated measures	(1) Refine, explore and provide preliminary testing of the multi-context approach in promoting strategy use across situations and increasing self-regulation, awareness and functional performance; (2) Participants demonstrated positive changes in self-regulatory skills and strategy use across tasks. Examination of individual participants revealed important, varying patterns of change in strategy use, learning transfer and self-awareness across intervention.
Cernich et al., 2010	Review; TBI	(1) Review of available evidence of cognition following TBI; (2) Recommendations: (i) Access to sub-acute rehabilitation that is holistic in nature and involves multi-disciplinary team to in work in an integrated fashion to support physical, cognitive and social skill retraining is vital to support positive outcome following TBI; (ii) Trials of medication to assist with attention, memory impairment appear well-supported by the available evidence; (iii) RCTs demonstrate utility of specific rehabilitation approached to attention retraining and retraining of executive function; (iv) Training in use of supportive devices to support individual's daily activities remains central to independent function.
Leon-Carrion et al., 2012	Acute severe TBI (*n* = 19); quantitative: observational	(1) Explore the course and timing of functional recovery in patients who have emerged from coma; (2) To achieve a good response and outcome nearing normalcy, a patient needs over 300 h of intensive rehabilitation.
Hayden et al., 2013	Acute-chronic mild-severe TBI (*n* = 1274); retrospective; quantitative	(1) Evaluate functional improvement after admission to post-acute rehabilitation; (2) Improved functioning after post-acute rehabilitation, regardless of severity of impairment or time since injury to admission to program. Rate of improvement greater for those admitted within 3 months of injury. Individuals with severe impairment demonstrated less improvement when admitted later in time after injury.

Acute, 0–3 months post-injury; post-acute, 3–12 months post-injury; chronic, greater than 12 months post-injury.

**Table 3:** **Detailed summary of articles included in “Post-discharge experiences and EE” scoping review**.

**Authors**	**Methods**	**(1) Main objectives and (2) Findings**
**POST-DISCHARGE FACTORS THAT INFLUENCE STIMULATION**		
Corrigan et al., 2004	Chronic mild-severe TBI (*n* = 1802); prospective; quantitative	(1) Provide population-based estimates of perceived needs following TBI and the prevalence of unmet needs; (2) Many reported still requiring help managing cognitive changes, emotional changes, and managing finances.
Staudenmayer et al., 2007	Post-acute severe TBI (*n* = 211); prospective; quantitative	(1) Determine whether there are specific types of functional deficits that disproportionately affect ethnic minorities after TBI; (2) Minorities demonstrated worse long-term functional outcome, less social and financial resources suggested as related/causal variables.
Shafi et al., 2007	Post-acute severe TBI (*n* = 344); prospective; quantitative	(1) Analyze whether racial or ethnic disparities exist in trauma care, specifically related to access to rehabilitation services and functional outcomes of patients with TBI; (2) Ethnic minorities less likely to be insured; more likely to have moderate- severe disability at follow-up. Data suggest insured patients less likely to be disabled, relationship strongest for private insurance.
Till et al., 2008	Post-acute moderate -severe TBI (*n* = 33); quantitative: observational cohort: 5, 12, 24 months post-injury	(1) Assess prospectively degree of post-acute long-term cognitive decline after TBI; (2) Amount of therapy received at 5 months post-injury significantly higher in group of non-decliners vs. decliners; individuals who were insured received more hours of therapy after discharge than those not insured.
Sander et al., 2009	Post-acute mild-severe TBI (*n* = 151); prospective; quantitative	(1) Determine contribution of race/ethnicity and income to community integration at approximately 6 months following TBI; (2) After controlling for age, education, injury severity, race/ethnicity, income made a significant contribution to variance in social integration, total score and scores on Belonging and Independent Participation scales of the Community Integral Measure. Lower income was associated with worse community integration.
Keightley et al., 2011	TBI, ABI + caregivers (*n* = 17); qualitative	(1) Explore barriers and enablers surrounding transition from health care to home community settings for Aboriginal clients recovering from ABI in northwestern Ontario; (2) Lack of awareness, education and resources acknowledged as key challenges to successful transitioning by clients and healthcare providers.
Sander et al., 2011	Post-acute mild-severe TBI (*n* = 167); prospective; mixed methods	(1) Investigate meaning of community integration in an ethnically diverse sample; (2) Financial issues, such as home ownership and insufficient funds, were perceived as contributing to decreased participation in the community.
Turner et al., 2009b	Post-acute mild-severe TBI, ABI + caregivers (*n* = 38); qualitative; pre-discharge, 1, 3 months post-discharge	(1) Explore people's lived experiences of reengagement in meaningful occupations during hospital-to-home transition phase after ABI; (2) Not being able to participate in desired occupations was source of stress and frustration. Many family caregivers reported participation in meaningful occupations was fundamental element of recovery gains. Other key elements: establishing routines or schedules and occupying one's time. Participation in meaningful occupations perceived to enhance functional recovery during transition.
**POST-DISCHARGE FACTORS THAT INFLUENCE ENGAGEMENT**		
Freeman, 1997	Severe TBI; Concept paper	(1) Explore methods used to establish a rehabilitation program in the home, the initial moves, the family dynamics, the advantages, and some of the programs required for the restoration of function of sensory, cognitive and motor abilities; (2) Family environment provides wide variety of activities, which are inclusive of person, guarantees provision of stimulation over a wide spectrum of inputs and activities.
Rotondi et al., 2007	Chronic moderate-severe TBI + caregivers (*n* = 185); qualitative	(1) Determine expressed needs of persons with TBI and their primary family caregivers; (2) Inadequate preparation of primary support persons and persons who experienced TBI for personality and behavioral sequelae prior to discharge from the hospital appeared to be a common complaint.
McCormack and Liddiard, 2009	Chronic severe TBI (*n* = 1); case study	(1) Examines a model of community rehabilitation; (2) Supportive and effective familial care system and specialist community interdisciplinary rehabilitation was effective in facilitating recovery.
Keightley et al., 2011	TBI, ABI + caregivers (*n* = 17); qualitative	(1) Explore barriers and enablers surrounding transition from health care to home community settings for Aboriginal clients recovering from ABI in north western Ontario; (2) Lack of awareness, education and resources acknowledged as key challenges to successful transitioning by clients and healthcare providers.
Sander et al., 2011	Post-acute mild-severe TBI (*n* = 167); prospective; mixed methods	(1) Investigate meaning of community integration in an ethnically diverse sample; (2) Feeling integrated into the community relates to aspects of the environment as much as to involvement in specific activities.
Turner et al., 2011b	Post-acute mild-severe TBI, ABI + caregivers (*n* = 38); qualitative; pre-discharge, 1, 3 months post-discharge	(1) Explore service and support needs of individuals with ABI and family caregivers during transition phase from hospital to home; (2) Individuals with ABI experience a range of service and support needs during the early transition phase, many of which are currently unmet. Findings also indicated that support for family caregivers and access to early community rehabilitation were the two areas in which participants most commonly reported experiencing unmet needs.
Rusconi and Turner-Stokes, 2003	Post-acute/chronic TBI, ABI, SCI (*n* = 53); quantitative: cross-sectional cohort survey	(1) Evaluate aftercare of patients discharged from specialist rehabilitation unit with respect to use of equipment and follow-up by therapy and care services and to assess change in dependency and care needs; (2) Many patients observed they were ill-prepared for sudden change from an intensive therapy programme on unit to a much less frequent and more self-reliant programme in community.
Rittman et al., 2004	Post-acute stroke + caregivers (*n* = 51); qualitative	(1) Evaluate the transition from hospital to home during the first month after discharge following acute stroke; (2) When routines are not re-established, survivors and caregivers experience more chaos, disruption during the transition. When talking about ways days are spent, most survivors describe problems with boredom and not having meaningful activities in their lives.
Turner et al., 2007	Chronic TBI, ABI + caregivers (*n* = 24); qualitative	(1) Explore transition experiences from hospital to home of a purposive sample of individuals with ABI; (2) Many participants found it difficult to locate and access suitable post-discharge therapy services. Friendship and social networks played important role during transition process. Post-discharge boredom, particularly during first 1–2 months at home, commonly expressed.
Turner et al., 2009b	Post-acute mild-severe TBI, ABI + caregivers (*n* = 38); qualitative; pre-discharge, 1, 3 months post-discharge	(1) Explore people's lived experiences of reengagement in meaningful occupations during hospital-to-home transition phase after ABI; (2) Not being able to participate in desired occupations was source of stress, frustration for many participants. Many family caregivers reported participation in meaningful occupations was fundamental element of recovery gains. Other key elements of transition success included establishing routines or schedules and occupying one's time. Results demonstrated participation in meaningful occupations was perceived to enhance functional recovery during transition; underscores importance of encouraging and facilitating participation in meaningful occupations.
Hoogerdijk et al., 2011	Chronic mild-severe TBI (*n* = 4); qualitative	(1) Better understand how individuals with TBI make sense of adaptation process and their performance of occupations within this process; (2) Results indicate adaptation process following TBI is a necessary struggle to gain new identity; facilitated by engagement in familiar occupations in familiar environments; a protracted learning process that continues long after rehabilitation ends; individual, situated.
Turner et al., 2011a	Post-acute mild-severe TBI, ABI + caregivers (*n* = 38); qualitative; pre-discharge, 1, 3 months post-discharge	(1) Explore perspectives of individuals with ABI and their family caregivers concerning recovery and adjustment during the early transition phase from hospital to home; (2) Findings highlight that while returning home was typically perceived to facilitate ongoing recovery, process of adjusting emotionally to life at home posed significant challenge for many participants during transition phase.
Nalder et al., 2012a	Post-acute moderate -severe TBI + caregivers (*n* = 210); prospective; quantitative: repeated measures: discharge, 1, 3, 6 months post-discharge	(1) Identify factors associated with perceived success of transition from hospital to home after TBI; (2) Greater perceived success of transition associated with higher levels of health-related quality of life, level of community integration, more severe injury. Sentinel events (e.g., returning to work, independent community access, changing living situation) associated with greater perceived success; financial strain, difficulty accessing therapy services associated with less success.
Nalder et al., 2012b	Post-acute moderate -severe TBI + caregivers (*n* = 210); prospective; quantitative: repeated measures: discharge, 1, 3, 6 months post-discharge	(1) Describe timing and factors associated with occurrence of sentinel events (financial strain, difficulty accessing therapy, return to work, accommodation change and independent transport use) during transition to community for individuals with TBI; (2) General positive sentinel events (e.g., regaining independence, returning to work) more likely experienced by individuals with higher levels of global functioning and psychosocial integration. Individuals with lower levels of functioning at greater risk of experiencing more negative sentinel events (e.g. financial strain). Individuals with more severe injury and poorer adjustment more likely to report difficulty accessing therapy.

Acute, 0–3 months post-injury; post-acute, 3–12 months post-injury; chronic, greater than 12 months post-injury.

## Environmental enrichment has beneficial effects on brain and behavior in animals and humans

### Defining environmental enrichment

A number of definitions have been proposed for EE, and it is often defined in relative terms. In animal studies, EEs (e.g., cages with running wheels, novel toys, several animals) are typically contrasted with standard or impoverished environments (e.g., cages with a single animal, and only the basic necessities for living). In these studies, researchers have stressed the importance of having cognitive, social and physical stimulation for environments to be considered enriched and a key property is the maintenance of novelty, for example through regularly changing toys and food (Diamond, 2001; Simpson and Kelly, 2011).

Kramer et al. (2004) have suggested that while numerous positive changes in cognitive functioning, neuroanatomy, and neurochemistry have been demonstrated as a result of exposure to EE in animals, we must examine the degree to which these findings translate to humans, and that an operational definition of EE applicable to humans needs to be established. Whereas novel toys and food, running wheels, and housing several animals together maps on well to cognitive, physical, and social stimulation in animals, identification of such concrete mappings in humans has proven more difficult. Subject factors, such as motivation and mental effort play a large role in reaping the benefits of EE. Thus, personality and earlier life experiences may influence engagement with the environment, such that what is a stimulating and engaging environment for one person may not be for another (Johansson, 2003). Thus, for humans, the definition of EE is more complex, addressing both the nature of the environment and factors that influence engagement with it.

These ideas of Kramer et al. (2004) are consistent with earlier work by Schooler (1987), who defined the related concept of environmental complexity as being determined by stimulus and demand characteristics. He theorized that greater diversity of stimuli in one's environment could lead to more options/plans of action to consider and decisions to make. When cognitive efforts are reinforced and rewarded, people are motivated to continue engaging in complex environments, which in turn enhances cognitive functioning. Thus, enhancing cognitive functioning further promotes participation in complex environments, illustrating a dynamic facet of EE and its benefits. Enrichment, therefore, reflects environmental complexity (e.g., opportunity to participate in different sports, clubs or social networks, and to engage in intellectually demanding activities), one's inclination to participate in the environment, and the frequency of participation.

Given the limitations in defining, manipulating and controlling EE in human studies, animal studies have arguably provided the most compelling evidence for the causal effects of EE.

### Benefits of environmental enrichment

#### Environmental enrichment in healthy animals

In 1947, Hebb examined if rats exposed to EE would improve behaviorally on problem-solving tasks, compared to a control group (Hebb, 1947). EE rats were free to roam his home while control rats were housed in standard laboratory cages. Hebb found performance was superior in the EE group. Since then, more systematically controlled studies have had similar findings.

Exposure to EE has been associated with increases in cognitive functioning, specifically improvements in response selectivity, learning ability, spatial and problem solving skills, memory, and processing speed (Mohammed et al., 1990; Rosenzweig and Bennett, 1996; Nilsson et al., 1999; Van Praag et al., 2000; Kobayashi et al., 2002; Milgram, 2003; Valero et al., 2011; Leger et al., 2012; Speisman et al., 2012; Yang et al., 2013). Reductions in boredom (Meagher and Mason, 2012) and frustration (Latham and Mason, 2010) have been demonstrated as well. The benefits of EE are also observable at cellular and molecular levels. There is evidence of increased neurogenesis, synaptogenesis and dendritic spine density in parts of the brain associated with memory and learning (i.e., hippocampus, dentate gyrus and cerebellar Purkinje cells) in response to EE (Kolb and Whishaw, 1998; Johansson, 2000, 2002; Van Praag et al., 2000; Churchill et al., 2002; Kempermann et al., 2002; Valero et al., 2011; Eckert and Abraham, 2012; Jung and Herms, 2012; Leger et al., 2012; Speisman et al., 2012; Fares et al., 2013; Yang et al., 2013). Increases in brain weight and cortical thickness have also been demonstrated, as have increases in the amount of nerve growth factor, brain derived neurotropic factor, myelination, acetylcholinesterase activity, neurotransmitters, glial proliferation, blood vessels (number and size), and protein synthesis (Rosenzweig, 1966; West and Greenough, 1972; Bennett et al., 1974; Mohammed et al., 1990; Rosenzweig and Bennett, 1996; Kolb and Whishaw, 1998; Van Praag et al., 2000; Diamond, 2001; Churchill et al., 2002; Pietropaolo et al., 2004; Will et al., 2004; Bennett et al., 2006; Hoffman et al., 2008; Gabriel et al., 2009a,b; Lores-Arnaiz et al., 2010; Qiu et al., 2011, 2012; Williamson et al., 2012; Fares et al., 2013; Vazquez-Sanroman et al., 2013; Yang et al., 2013).

In examining properties of EE that produce beneficial effects, factors such as *intensity* and *duration* emerge as critical for reaping the most benefits. Bennett et al. (2006) examined the effects of long-term continuous EE on memory performance in aged mice. Mice received a 10-week treatment of either 5 min of daily handling housed in impoverished environments (i.e., clear plastic shoe boxes), 3 h of daily exposure to a basic EE (i.e., small bin with fresh bedding, a running wheel, a plastic rodent dwelling, a plastic tube for climbing, a few toys), or continuous complex EE (i.e., larger bin to allow for more mice to be housed together, and for larger and more complex objects). Continuously enriched older mice performed significantly better than other groups of aged mice in spatial memory tasks, and were indistinguishable from younger control mice in their performance.

Amaral et al. (2008) examined durations of EE in mice and influences on behavioral changes (open field habituation and locomotion). They compared mice exposed to 1, 4, and 8 weeks of EE. While mice exposed to 4 and 8 weeks showed behavioral effects, the 1-week group did not. Furthermore, mice in the 4-week exposure group demonstrated effects lasting 2 months post-EE, but only mice in the 8-week EE group demonstrated effects lasting up to 6 months. The authors concluded that *(1) a minimum EE period is necessary to induce beneficial behavioral effects, (2) the effect of EE can persist at least partially for many months after its cessation, and (3) the degree of persistence is greater in animals exposed to longer durations of EE*. Relevant to the discharge environment of humans and the importance of ongoing therapy/stimulation, these findings showed that the benefits of EE are lost when animals are removed from enriched environments.

#### Environmental enrichment in healthy humans

Scarmeas and Stern (2003) undertook a review of the relationship between lifestyle and cognitive reserve, which they defined as the degree to which the brain can create and use networks or cognitive paradigms that are more efficient or flexible, and thus less susceptible to disruption. They asked whether exercising the brain had the same healthy effects as exercising the body. They found that greater participation in intellectual and social leisure activities was associated with less cognitive decline in healthy older adults and a lower incidence of dementia. These important “use it or lose it” findings support the need for EE in the sub-acute and chronic stages of TBI to avert decline. The authors proposed that leisure activities and an active lifestyle might buffer against cognitive decline by: (1) increasing synaptic density, which results in more efficient cognitive functioning of unaffected neurons; (2) more efficient use of the same brain networks; and (3) more efficient use of alternative brain networks. They also found that although it is a very active area of research, our understanding of the specific active ingredients that buffer against cognitive decline has not been fully elucidated.

In addition to studies examining how mentally active lifestyles are associated with cognitive benefits (Gribbin et al., 1980; Pushkar et al., 1997; Wang et al., 2002; Wilson et al., 2002, 2003; Crowe et al., 2003; Verghese et al., 2003; Newson and Kemps, 2005; Fujiwara et al., 2009), studies have also illustrated the benefits of maintaining physically active (Clarkson-Smith and Hartley, 1989; Schuit et al., 2001; Pignatti et al., 2002; Voss et al., 2011; Xu et al., 2011) and socially active lifestyles (Bassuk et al., 1999; Fratiglioni et al., 2000; Mahoney et al., 2000; Seeman et al., 2001). However, they are correlational in design and leave open the possibility that higher functioning people seek out the continuous exposure.

A number of further studies have shown the benefits of EE for healthy older adults (Huppert, 1991; Christensen and Mackinnon, 1993; Davidson and Bar-Yam, 2006; Sampson et al., 2009; Marioni et al., 2012a,b; Paillard-Borg et al., 2012). Winocur and Moscovitch (1990) compared cognitive functioning in community-dwelling and institutionalized older adults. After controlling for variables such as IQ, age, and health, they found that community-dwelling older adults had higher levels of cognitive functioning. They also identified a subgroup of high-functioning institutionalized older adults who performed similarly to the community-dwelling group. For this subgroup, they postulated that cognitive functioning may have been influenced by adjustment to institutional life, such that more social activity was correlated with better cognitive functioning, as was found in a prior study (Winocur et al., 1987). Winocur and Moscovitch suggested that lower cognitive functioning observed in the institutionalized group might be a result of an under-stimulating environment. This interpretation is also consistent with the position of Schooler and Mulatu (2001), who suggested that even if people have higher levels of education, it is the *continued practice* in complex activities that maintains their cognitive abilities—speaking directly to the thesis of this paper.

A number of studies have examined the benefits of continuous exposure (Blackerby, 1990; Spivack et al., 1992; Willer et al., 1999; De Weerdt et al., 2000; Shiel et al., 2001; Zhu et al., 2001; Cifu et al., 2003; De Wit et al., 2005), and interventions that incorporate many elements of EE, such as cognitive, social, and physical stimulation (Hayslip et al., 1995; Neely and Backman, 1995; Fasotti et al., 2000; Powell et al., 2002; Vance and Johns, 2002; Gunther et al., 2003; Rath et al., 2003; Noice and Noice, 2004; Van De Winckel et al., 2004; Green et al., 2006; Stine-Morrow et al., 2007; Spikman et al., 2009). These types of interventions can be contrasted with interventions involving manipulations over a discrete period of time that are designed to enhance a specific cognitive domain or skill. The former have broadly led to more generalizable gains, both cognitively and physically.

#### Brain-injured animals and environmental enrichment

Brain-injured animals show clear-cut benefits from exposure to EE. Studies comparing recovery in brain-injured animals reared in EEs to those reared in standard environments have demonstrated increased neurogenesis, upregulation of neurotrophic factors, increased neuronal survival, increased afferent innervation, as well as reductions in spontaneous apoptosis and infarct size (Van Praag et al., 2000; Dobrossy and Dunnett, 2001, 2004; Passineau et al., 2001; Gobbo and O'Mara, 2004; Will et al., 2004; Pereira et al., 2007; Hoffman et al., 2008; Sozda et al., 2010; Sun et al., 2010; Monaco et al., 2013; Shin et al., 2013). Superior performance on tasks of learning and memory, and spatial reference has also been found (Hamm et al., 1996; Johansson and Ohlsson, 1996; Passineau et al., 2001; Gobbo and O'Mara, 2004; Will et al., 2004; Lippert-Gruener et al., 2007; Pereira et al., 2007; Hoffman et al., 2008; Sozda et al., 2010; Monaco et al., 2013). Hamm et al. (1996) compared cognitive functioning in brain-injured and sham-brain-injured rats housed in EEs to those housed in standard environments. Rats housed in EEs were in social groups and had access to a variety of foods, novel toys, scented objects, and a running wheel. At 15 days post-injury, brain-injured rats exposed to EE performed significantly better than brain-injured rats in standard environments. Interestingly, they also found that brain-injured rats recovering in EE performed just as well as non-brain injured animals.

In an older but seminal study by Johansson and Ohlsson (1996), brain-injured rats recovering in comprehensive EE (i.e., cognitive, social, and physical enrichment) were compared to those recovering in either enriched social environments or enriched exercise environments. Rats in the EE group were placed in one cage with several other rats, and novel toys and objects that promoted exercise, whereas rats in the social-interaction group were caged with other rats without objects or toys, and rats in the exercise group were individually caged with access to a running wheel. The social-interaction group improved more than the exercise group; however, the EE group improved more than either group. More recently, Hoffman et al. (2008) demonstrated the importance of timing and duration of EE, comparing brain-injured rats exposed to continuous EE to those in early and temporary EE (immediately post-injury lasting 1 week), late EE (commencing 1 week post-injury) and no EE. The continuous EE group not only had significantly less hippocampal cell loss, but also performed better on motor and cognitive tests. Interestingly, they found an advantage of continuous and *early* EE for motor tasks, but an advantage of continuous and *delayed* EE for cognitive tasks. The benefits of increased EE-exposure in brain-injured rats have since been replicated (De Witt et al., 2011; Matter et al., 2010), and, moreover, shown to last for up to 6 months (Cheng et al., 2012).

These studies provide evidence that EE help animals to recover functions to levels that make them indistinguishable from healthy controls, and that *continual* EE is necessary to *maintain* both neural and cognitive improvements. The benefits of EE are substantial, but not permanent, thus highlighting the importance of ongoing stimulation. These findings are particularly relevant to the post-discharge environment, and the factors may contribute to decline.

#### Brain-injured humans and environmental enrichment

EE interventions for brain-injured humans can be grouped into two broad types: those that provide increased hours and duration of therapy (without prescription of specific content of therapies) and those that address discrete impairments and focus on improvement of a specific skill. Note that in clinical practice, the latter might be referred to as “training” rather than EE. However, we include them here as they entail increased cognitive stimulation.

While EE intervention designs have not been as comprehensive in human TBI studies, the effects of some of the classical properties of EE, such as novelty, intensity, and duration, have been examined. However, much like in healthy individuals, interventions for TBI survivors aimed at training specific skills have often resulted in poor generalization to everyday performance (Ruff et al., 1991; Cicerone et al., 2000, 2005; Sohlberg et al., 2000; Park and Ingles, 2001; Boman et al., 2004; Kennedy et al., 2008). In contrast to skills training, Cicerone et al. (2000) have suggested that cognitive rehabilitation should not focus exclusively on remediation of impairments, but should reduce disability and help restore social role functioning. In an earlier framework, Toglia (1991) proposed a “multi-context” treatment approach to enhance generalizability that contained hallmarks of what later became EE: namely, varied stimulation and environments that are meaningful to the person that thereby enhance engagement. Interventions that have incorporated such components have demonstrated better generalizability, as well as improvements in community integration (Fasotti et al., 2000; Powell et al., 2002; Vance and Johns, 2002; Rath et al., 2003; Van De Winckel et al., 2004; Toglia et al., 2010; Leon-Carrion et al., 2012).

The second broad category of interventions that are more intensive have also resulted in significant functional gains (Willer et al., 1999; Cicerone et al., 2004). Cicerone et al. (2004) compared the effectiveness of an intensive cognitive rehabilitation program to standard neurorehabilitation for persons with TBI. Participants in the intensive program demonstrated greater improvement in cognitive function (composite score based on attention, speed of processing, memory and executive function test scores) as well as greater improvement in community integration. Increasing hours of therapy has also led to greater functional and cognitive gains and faster recoveries (Blackerby, 1990; Spivack et al., 1992; De Weerdt et al., 2000; Shiel et al., 2001; Zhu et al., 2001; Slade et al., 2002; Cifu et al., 2003; De Wit et al., 2005; Zhu et al., 2007). Turner-Stokes et al.'s (2005) review of multi-disciplinary rehabilitation for brain injury recommended that “intensive intervention appears to lead to earlier gains.” Therefore, they concluded that “Patients discharged from in-patient rehabilitation should have access to out-patient or community-based services appropriate to their needs.” Cernich et al. (2010) provided a similar recommendation in their review of cognitive rehabilitation following TBI. Recently, Hayden et al. (2013) provided support for this recommendation, demonstrating functional gains for TBI survivors receiving rehabilitation services in the post-acute phase after brain injury.

### Absence or reduction of environmental enrichment

If exposure to EE is beneficial at both behavioral and neural levels, it logically follows that the absence or reduction of EE would lead to poorer behavioral and neural health. Experience-dependent plasticity refers to changes in the brain that result from behavioral experiences. While stimulation can enhance or maintain neural pathways, the absence of stimulation can depress or weaken them with associated loss of a function that was previously acquired or mastered (Rubinov et al., 2009; Warraich and Kleim, 2010). Overall, there appears to be a growing consensus that maintaining and increasing neuroplasticity of the brain depends on continual and intensive cognitive, physical, and social stimulation (Kleim and Jones, 2008). TBI survivors transitioning from a rehabilitation setting to a home setting are therefore vulnerable to reversal of those gains made in the early and intensive therapeutic setting.

This assertion is supported by the flipside of EE experiments: animals that are exposed to less stimulating or impoverished environments do not fare as well. As described above, mice with less EE exposure have lower performances on spatial memory tasks, as compared to continuously enriched mice (Bennett et al., 2006), and rats that received less EE exposure maintain benefits for a shorter period of time (Amaral et al., 2008). Similarly, brain injured rats recovering in impoverished environments show poorer recovery of cognitive and motor functioning (Hamm et al., 1996; Hoffman et al., 2008; Matter et al., 2010; De Witt et al., 2011) and do not maintain benefits gained from short-term EE (Matter et al., 2010; De Witt et al., 2011; Cheng et al., 2012).

Furthermore, there is evidence that effects of environmental conditions are reversible (Bernstein, 1972; Winocur, 1998). Winocur (1998) demonstrated that when he transferred rats from impoverished to enriched environments, they improved significantly, whereas rats transferred from enriched and standard environments to impoverished ones declined. Similarly, more sedentary lifestyles have been linked to disease and disability, and overall poorer health (Huppert, 1991; Pushkar et al., 1997; Mackinnor et al., 2003; Salthouse, 2006; Shors et al., 2012).

### Environmental enrichment, post-acute decline and sub-acute atrophy

As therapies diminish in frequency over time (e.g., when patients move from in-patient neurorehabilitation to the home or a chronic-care facility), the amount of EE may also lessen. In the context of chronic TBI, Turner and Green (2008) examined the principles of negative neuroplasticity (maladaptive morphological changes to the brain in response to environmental factors), as described by Mahncke et al. (2006a,b), in the context of normal aging. These are: (1) reduced schedules of activity, (2) noisy processing in peripheral and central sensory systems, (3) alterations in neuromodulary control, and (4) negative learning. They suggest that a similar downward spiral of negative neuroplastic change may undermine long-term outcome in TBI due to similar environmental changes (as well as physiological changes) that result in withdrawal from communities and social networks, resulting in reduced stimulation. Such changes are withdrawal from the workforce (or school), physical losses that preclude travel, and perceptual and cognitive decrements that compromise communication.

As previously noted, in the case where neural pathways are under-stimulated, it is possible to lose the function that was acquired or mastered (Rubinov et al., 2009; Warraich and Kleim, 2010). Findings of increased neural representation after training (Cirillo et al., 2011; Cardinali et al., 2012) and different brain activation when comparing exposure vs. deprivation of stimulation (Klinge et al., 2012) provide evidence for experience-dependent neuroplasticity. This raises the question whether under-stimulation of neural pathways, due to disuse, may be responsible for post-acute neural degeneration seen in TBI survivors. If so, EE exposure may play a role in minimizing, or averting, this problem.

With respect to neural degeneration, mechanisms of apoptosis have been modeled in animals to understand the cascade of events that takes place after TBI (Raghupathi et al., 2000; Keane et al., 2001; Raghupathi, 2004). Coulson et al. (2004) provided support for their hypothesis that synaptic input may be the key to regulating neuronal survival and death pathways following neurotrauma. Synaptic activity regulates expression levels of neurotrophins and facilitates growth factor signaling. There is evidence that EE exposure enhances nerve growth factor and brain-derived neurotropic factor in animals (Clarkson-Smith and Hartley, 1989; Blackerby, 1990; Bassuk et al., 1999; De Weerdt et al., 2000; Mahoney et al., 2000; Wang et al., 2002; Wilson et al., 2002; Cifu et al., 2003; Johansson, 2003; Rath et al., 2003; Spikman et al., 2009). As well, animal studies have demonstrated that EE can enhance neurogenesis and attenuate apoptosis in the injured brain (Van Praag et al., 2000; Dobrossy and Dunnett, 2001, 2004; Passineau et al., 2001; Gobbo and O'Mara, 2004; Will et al., 2004; Pereira et al., 2007; Hoffman et al., 2008). Lastly, neurons deprived of synaptic input can be rescued from death by an increased supply of growth factors (Coulson et al., 2004). Taken together, these findings offer the possibility that EE can help to prevent neuronal death.

The relation between changes in brain structure and neuropsychological performance has also been recently explored (Farbota et al., 2012), providing further evidence that as we see declines at a neural levels, we also see parallel declines in cognitive functioning. Recent work from our laboratory has demonstrated that in TBI survivors, more engagement in EE, defined as participation in activities involving cognitive, physical, and social demands, is correlated with less hippocampal volume loss in the post-acute stages (Miller and Green, in press). While these findings are correlational, they provide preliminary evidence of the positive impact that an enriched environment can have in TBI survivors.

The research reviewed within this section of the scoping review supports that EE improves outcomes and that a dearth of EE results in poorer outcomes. Studies demonstrate continued and repeated EE exposure (Blackerby, 1990; Spivack et al., 1992; De Weerdt et al., 2000; Schooler and Mulatu, 2001; Shiel et al., 2001; Zhu et al., 2001, 2007; Cifu et al., 2003; De Wit et al., 2005; Amaral et al., 2008), which provides intensive cognitive, social, and physical stimulation is necessary to induce beneficial effects (Willer et al., 1999; Cicerone et al., 2004; Bennett et al., 2006). Furthermore, if this exposure is reduced or removed, the benefits will not be maintained and may diminish (Bernstein, 1972; Winocur, 1998; Cheng et al., 2012). Synaptic input may be necessary to maintain and strengthen neural pathways and connections, particularly those at risk after brain injury (Coulson et al., 2004; Turner and Green, 2008). Moreover, this research supports our contention that for the subset of people with moderate-severe TBI that show post-acute cognitive decline and neural deterioration in the post-acute phase, there may be some environmental variables that contribute to their negative outcomes.

## Post-discharge experiences and environmental enrichment

In the first section, we discussed findings revealing that the brain atrophies and that cognitive abilities decline in the post-acute stages of brain injury. In the second section, we discussed findings that demonstrate that EE can alter the brain (both healthy and damaged), and perhaps most importantly that it has the potential to minimize atrophy after brain injury. Moreover, a mechanism of disuse-mediated atrophy has been proposed (i.e., synaptic input and neuronal survival) (Coulson et al., 2004).

A critical question, then, is to what extent do post-discharge factors reflect EE? This is important because it is possible that the post-discharge environment may be un-enriched, and this would in theory exacerbate/lead to post-acute cognitive and neural declines. Thus, it is of value to explore the factors that influence the environments in which people with brain injuries return with respect to level of enrichment, so that factors can be adjusted to provide *optimal levels of EE*. In the current section, we will discuss how post-discharge variables are conceptually related to EE and we will also discuss findings that show how these examples of EE (or lack thereof) correspond to human TBI outcomes.

### What is environmental enrichment in the post-discharge environment?

Continued participation in environments that are challenging, yet at levels that allow people to participate and remain motivated to do so, is the crux of EE. In the section “Environmental Enrichment in Healthy Humans” we summarized findings demonstrating that people who (1) attend more social events, (2) are more physically active, and (3) engage in activities with continuous novelty (e.g., learning a new language, playing bridge) are mentally healthier. While demographic variables (i.e., age, pre-morbid intelligence, level of education) influence outcome after brain injury (Ruff et al., 1991; Green et al., 2008), environmental variables (e.g., access to insurance coverage) also play a vital role in recovery, as will be discussed. We contend that these variables map onto EE in that they provide cognitive, social, and physical stimulation through access to therapy, community resources, fostering return to meaningful occupations, and encouraging engagement in their environments.

### Post-discharge environmental factors that influence mental and physical stimulation

A number of factors influence the degree of cognitive, social, and physical stimulation TBI survivors experience either directly (e.g., presence of community resources such as a support groups or fitness centers) or indirectly (e.g., by influencing access to therapy). These include insurance, financial support, social support, and community resources.

#### Insurance

As recommended by Turner-Stokes et al. (2005), after discharge from in-patient rehabilitation, TBI survivors should have access to out-patient or community-based services appropriate to their needs to facilitate the recovery process. Till et al. (2008) demonstrated that post-acute cognitive decline was negatively correlated with hours of therapy, which was associated with insurance coverage. It has been widely demonstrated that individuals who have insurance coverage receive more access to therapy after discharge than those who do not (Pressman, 2007; Heffernan et al., 2011; Chen et al., 2012; Lundqvist and Samuelsson, 2012). Those that are insured have better access to post-acute medical care, which includes physical, occupational, and cognitive therapies, as well as home health and nursing needs, modification of living environment, vocational training, and job retraining (Shafi et al., 2007). In line with these findings, Shafi et al. (2007) found that ethnic minorities were less likely to be insured and more likely to have moderate to severe disability at follow-up.

#### Financial support

Diminished financial resources may reduce opportunities for accessing EEs. For example, Sander et al. (2009) reported that after controlling for age, education, injury severity, and race/ethnicity, income made a significant contribution to the variance in social integration, and in a more recent study, Sander et al. (2011) reported that TBI survivors perceived financial issues (e.g., home ownership, insufficient funds), as contributing to decreased participation in the community. Similar to Shafi et al. (2007) discussed previously, Staudenmayer et al. (2007) examined ethnic disparities in long-term functional outcomes after TBI. They concluded that less social and financial resources were likely implicated as an explanation. Additionally, in a study examining perceived needs after brain injury, many TBI survivors reported still requiring help managing money 1 year post-injury (Corrigan et al., 2004), which may compound the challenges of diminished finances after TBI. Increased financial burden can mean less access to expensive resources (e.g., therapy, participating in social activities, transportation).

#### Social support

Logically, a supportive social network influences participation in therapy (Sander et al., 2009; Turner et al., 2009b; Keightley et al., 2011), for example by providing transportation, accompaniment to therapies, supervision for recommended regimens for which safety is of concern (e.g., a gym program). Turner et al. (2007) found that those who had more supportive and closer networks at home had better transitions from the hospital setting to the home environment, as this was related to more access to social activities and transportation outside the home.

### Post-discharge factors that influence engagement

Without private insurance, and with less social and financial resources, TBI survivors may be less able to engage in stimulating activities to facilitate recovery. However, as we will discuss, there are also factors that are purported to influence the level of engagement in the post-discharge environment, such as the amount of family support and patient education provided, as well as the structure and routine present in their home environment.

#### Family support

In Freeman's (1997) review of community-based rehabilitation for TBI survivors, he suggested that the level of care in the home setting, with a strong support network, can play a major role in successful rehabilitation. The family environment may provide a wide variety of activities that are inclusive, stimulating, and meaningful to the individual. Importantly, these are all properties found to be critical for experience-dependent plasticity (Kleim and Jones, 2008) and generalization of relearnt skills (Toglia, 1991). In a case study by McCormack and Liddiard (2009), a TBI survivor receiving community rehabilitation was followed for 3 years. The authors concluded that the supportive and effective care system in his family facilitated his recovery following severe TBI. His progress was attributed to active familial involvement that fostered goal setting and carry-over between sessions. Furthermore, learning took place in his home environment, increasing his ability to generalize skills. The authors concluded that these findings “add further weight … to the thesis that, with the right support, there is no place like home.”

#### Post-discharge information and education

Several studies have indicated that the most often-reported barrier is adequate preparation prior to discharge (Rotondi et al., 2007; Sander et al., 2009; Keightley et al., 2011; Sander et al., 2011; Turner et al., 2011b). Many TBI survivors and their caregivers report that they were not given enough information regarding brain injuries (e.g., behavioral sequelae), how to access community resources (e.g., rehabilitation, emotional support, respite services), or how to access or implement home therapies (Rotondi et al., 2007; Sander et al., 2009, 2011; Keightley et al., 2011; Turner et al., 2011b). Corrigan et al. (2004) reported that TBI survivors who initially reported requiring help participating in recreation still did not have their perceived needs (viewed as a measure of quality of life) met 1 year post-injury. Likewise, Sander et al. (2011) reported that for TBI survivors, feeling more integrated into the community was related to greater participation in their environments. Without receiving appropriate assistance and information/educational resources to facilitate the post-discharge process, successful community integration will continue to be a challenge.

#### Routine and schedules

Many studies document that TBI survivors report feeling ill-prepared for the transition from hospital to home (Rusconi and Turner-Stokes, 2003). Several qualitative studies have explored the transition home, and the barriers or difficulties that TBI survivors experience (Rittman et al., 2004; Turner et al., 2007, 2009b, 2011a). Rittman et al. (2004) found that, post-discharge, commonly reported problems were increased idle time, boredom, and little-to-no engagement in meaningful activities. Survivors often reported, “… finding something new every day that they couldn't do …” What appears to influence the above is routine, or lack thereof. When routines were not re-established, survivors and caregivers experienced more chaos and disruption in the transition home. Turner et al. (2011a) examined perspectives concerning recovery and adjustment during the transition phase from hospital to home, and found that the process of adjusting emotionally to life at home posed significant challenges for many brain-injury survivors during this phase.

Turner et al.'s (2007) qualitative study of brain injury survivors' experiences with the transition from hospital to home made similar observations in terms of difficulty re-engaging in meaningful activities and occupations. However, those who were able to create a routine or structure once at home had better transitions. Essentially, this facilitated participation in their environments by providing organization in their daily schedules, which had been previously often provided for them in the rehabilitation setting.

Turner et al. (2009b) extended their work by examining re-engagement in meaningful occupations during the transition from hospital to home in brain-injury survivors. Many survivors reported that recovery sped up at home, and caregivers frequently attributed this to key elements such as creating routines and schedules, which enabled participation in meaningful activities. However, for those that did not have such experiences, not being able to participate in desired occupations led to stress and frustration. Turner et al. (2009b) found that “for some participants, the impact of this change in environment, coupled with the reality of not being able to engage in their desired occupations, led to a relatively unproductive lifestyle in which the main activity of their day involved little more than watching television or playing computer games.” In Hoogerdijk et al.'s (2011) study of identity after TBI, the authors suggested that the adaptation process is a necessary struggle to gain new identity and it is facilitated by engagement in familiar occupations in familiar environments. In line with this finding, Nalder et al. (2012a,b) reported that TBI survivors that were able to reengage in meaningful occupations had greater perceived success of the transition from hospital to home. Reengagement in meaningful occupations was often experienced by individuals with higher levels of global functioning and psychosocial integration.

### Summary of post-discharge experiences and environmental enrichmment

The research presented in this section of the scoping review provides evidence that there are variables in the post-discharge environment that influence the amount stimulation and other variables that influence the level of engagement in EE. Largely, evidence suggests that those with better access and resources that map onto EE (i.e., insurance coverage, financial and social support) and those that are in environments that encourage participation (i.e., strong familial support, access to educational resources, more structure) have better functional outcomes.

## Discussion

The most direct (preliminary) evidence of the benefits of EE in the post-acute stages of recovery comes from work by Miller and Green (in press), where more engagement in enriched environments, defined as participation in activities involving cognitive, physical, and social demands, was correlated with less hippocampal volume loss. Further direct evidence of comes from Till et al.'s (2008) finding of a relationship between hours of therapy at 5 months post-injury and degree of cognitive decline, which the authors speculated was ay be due in part to lack of access to complex and enriched environments.

As we have discussed, a large corpus of studies have demonstrated the benefits of EE in healthy and brain-injured animals. There is evidence of neuroplastic change after EE exposure, with beneficial changes to neuroanatomy and neurochemistry (Kramer et al., 2004). These studies further support Coulson et al.'s (2004) hypothesis that synaptic input is critical to offset atrophy, in that increased input regulates neuronal survival, and prevents or attenuates apoptosis. This is of particular relevance to the neural decline observed in the post-acute stages after TBI. Turner and Green's (2008) re-formulation of the work of Mahncke et al. (2006a,b) in healthy older adults—that negative neuroplasticity (i.e., reduced activity schedules) may undermine long-term outcome in TBI—is also in accord with the hypothesis that EE may indeed play an important role in recovery.

In humans, engaging in cognitively, socially, and physically stimulating activities is related to better cognitive functioning in younger and older adults. Studies have also demonstrated that therapies for TBI survivors delivering continuous and intensified interventions result in better recovery at all levels of analysis, as well as better functional gains. These therapies have critical elements of EE, such as novelty, intensity, and prolonged periods of engagement in meaningful activities.

According to Schooler's (1987) model of environmental complexity, when cognitive efforts are reinforced and rewarded, people are motivated to continue engaging in complex environments, which in turn enhances cognitive functioning. However, TBI survivors may show reduced participation in complex environments, due to cognitive impairment, lack of psychological support/facilitation, or lack of resources, and thereby fail to obtain the emotional and neuromodulatory rewards for engaging. These conditions create a downward spiral of negative neuroplasticity (Turner and Green, 2008). Animal literature examining EE has provided critical evidence for the benefits of maintaining high levels of enrichment and stimulation post-discharge (Hamm et al., 1996; Bennett et al., 2006; Amaral et al., 2008; Hoffman et al., 2008). Findings that support the “Use it or Lose it” theory also suggest that continued engagement in mentally effortful activities is necessary to maintain cognitive functioning (Huppert, 1991; Pushkar et al., 1997; Mackinnor et al., 2003; Salthouse, 2006; Shors et al., 2012), and that an individual's environment (e.g., stressful or under-stimulating) can influence level of activity (Winocur and Moscovitch, 1990; Winocur, 1998). While TBI survivors may benefit from capacity-enhancing and intensified therapies provided in hospital, animal models have demonstrated that the effects of this enrichment will only continue to persist with longer exposure periods (Amaral et al., 2008). Furthermore, transitioning from a more stimulating/complex environment to a lesser one may result in a loss of benefits (Winocur, 1998).

The aim of this paper was to examine the question whether EE in the sub-acute and chronic stages of injury can influence progressive neural and cognitive decline. The aim of the first section was to establish the role of EE in long-term outcomes, and in particular, in offsetting decline; the aim of the second section was to illustrate that in the post-acute stages of injury, a number of factors influence the degree of EE. We have argued that post-discharge environments map onto conventional variables considered to create EE. Access to rehabilitative, social, and community resources, as well as strong support networks, provides essential cognitive, social, and physical stimulation. Moreover, these factors have shown to influence outcomes.

While there is strong evidence to suggest that TBI may be a progressive injury, more research needs to be conducted to further elucidate the role of EE in influencing this decline. It is important to note that the exact active ingredients of current interventions that incorporate multi-context environments and higher intensity therapies are still unclear, and how they compare to therapies that incorporate all elements of EE is still unknown. Furthermore, studies are needed that examine whether there is a correlation between post-acute brain changes and declines in function, as well as the type of environments that TBI survivors return to post-discharge. Turner et al. (2008) recommended that research is needed to explore the transition home after brain injury in order to “(1) Develop a comprehensive theoretical framework of the transition phase; and (2) facilitate both the validation of current intervention strategies and the development of innovative/tailored intervention approaches.”

As reported by caregivers and TBI survivors, creating a routine and structure around daily activities facilitated engagement. Furthermore, activities that are meaningful and at an appropriate level (e.g., tailored to the individuals specific impairments) to facilitate participation are critical. Researchers that have examined the needs of TBI survivors and their caregivers in the post-acute phase have recommended increased education provided by health care institutions would be beneficial to ease the transition from hospital to home (Turner et al., 2007, 2009a, 2011b; Arango-Lasprilla et al., 2011). Based on the results of our scoping review, we suggest that more resources and guidelines on how to create structure and daily routines, as well as information regarding self-administered therapies and activities for the individual and their caregiver, would be beneficial. Further rehabilitation support during the post-acute stages of recovery, such as home assessments or on-going therapy maintenance, would also be optimal. Gan et al. (2010) examined the support needs after brain injury and made several recommendations for support services. They suggested that family systems-based services should be accessible, life-long, individualized and flexible, as well as efficient, and that support system-based services should include proper diagnoses, incorporate a multi-component approach, provide brain-injury-specific services that are responsive to needs with proactive follow-ups, and importantly, provide continuous education. The benefits of social peer-mentoring programs as an intervention to enhance improvements in social integration for TBI survivors has also shown promise in recent studies and is currently being further investigated (Struchen et al., 2011; Hanks et al., 2012).

TBI survivors often fail to return to pre-injury levels of cognitive function, as well as employment and income, and these factors have been shown to be related with life satisfaction, perceived quality of life, depression and social isolation (Christensen et al., 2008; Eriksson et al., 2009; Hawthorne et al., 2009; Resch et al., 2009; Shigaki et al., 2009; Strandberg, 2009; Tsaousides et al., 2009). Assuming that post-acute atrophy contributes to the failure to return to pre-injury levels of cognitive function and thus successful community integration, then the clinical consequences are notable.

While the elements that comprise EE may be different from one person to another, it is evident that engagement in such environments is beneficial at both the cognitive and neural level, and thus it is plausible that EE can offset post-acute deterioration. Providing information and support to ease the transition home, information about the benefits of an enriched environment and guidelines on how to successfully participate in enriched settings, may be key elements in improving recovery. Most importantly, it may be a critical factor to facilitate successful community integration.

### Conflict of interest statement

The authors declare that the research was conducted in the absence of any commercial or financial relationships that could be construed as a potential conflict of interest.

## References

[B1] Amaral O. B. Vargas R. S. Hansel G. Izquierdo I. Souza D. O. (2008). Duration of environmental enrichment influences the magnitude and persistence of its behavioral effects on mice. Physiol. Behav. 93, 388–394 10.1016/j.physbeh.2007.09.009 17949760

[B2] Arango-Lasprilla J. C. Nicholls E. Villasenor Cabrera T. Drew A. Jimenez-Maldonado M. Martinez-Cortes M. L. (2011). Health-related quality of life in caregivers of individuals with traumatic brain injury from Guadalajara, Mexico. J. Rehabil. Med. 43, 983–986 10.2340/16501977-0883 22031343

[B3] Arksey H. O'Malley L. (2005). Scoping studies: towards a methodological framework. Int. J. Soc. Res. Methodol. 8, 19–32

[B4] Basso A. (1989). Spontaneous recovery and language rehabilitation in Cognitive Approaches in Neuropsychological Rehabilitation, eds Seron X. Deloche G. (Hillsdale, NJ: Lawrence Erlbaum), 17–37

[B5] Bassuk S. Glass T. Berkman L. (1999). Social disengagement and incident cognitive decline in community-dwelling elderly persons. Ann. Intern. Med. 131, 165–173 10428732 10.7326/0003-4819-131-3-199908030-00002

[B6] Bendlin B. B. Ries M. L. Lazar M. Alexander A. L. Dempsey R. J. Rowley H. A. (2008). Longitudinal changes in patients with traumatic brain injury assessed with diffusion-tensor and volumetric imaging. Neuroimage 42, 503–514 10.1016/j.neuroimage.2008.04.254 18556217 PMC2613482

[B7] Bennett E. L. Rosenzweig M. R. Diamond M. C. Morimoto H. Hebert M. (1974). Effects of successive environments on brain measures. Physiol. Behav. 12, 621–631 4824387 10.1016/0031-9384(74)90212-1

[B8] Bennett J. McRae P. Levy L. Frick K. (2006). Long-term continuous, but not daily, environmental enrichment reduces spatial memory decline in aged male mice. Neurobiol. Learn. Mem. 85, 139–152 10.1016/j.nlm.2005.09.003 16256380

[B9] Bernstein L. (1972). The reversibility of learning deficits in early environmentally restricted rats as a function of amount of experience in later life. J. Psychosom. Res. 16, 71–73 5062049 10.1016/0022-3999(72)90027-x

[B10] Bigler E. D. Blatter D. D. Anderson C. V. Johnson S. C. Gale S. D. Hopkins R. O. (1997). Hippocampal volume in normal aging and traumatic brain injury. AJNR Am. J. Neuroradiol. 18, 11–23 9010515 PMC8337859

[B11] Blackerby W. F. (1990). Intensity of rehabilitation and length of stay. Brain Inj. 4, 167–173 2331546 10.3109/02699059009026162

[B12] Blatter D. D. Bigler E. D. Gale S. D. Johnson S. C. Anderson C. V. Burnett B. M. (1997). MR-based brain and cerebrospinal fluid measurement after traumatic brain injury: correlation with neuropsychological outcome. AJNR Am. J. Neuroradiol. 18, 1–10 9010514 PMC8337869

[B13] Boman I. L. Lindstedt M. Hemmingsson H. Bartfai A. (2004). Cognitive training in home environment. Brain Inj. 18, 985–995 10.1080/02699050410001672396 15370898

[B14] Bruns J. Jr. Hauser W. A. (2003). The epidemiology of traumatic brain injury: a review. Epilepsia 44 Suppl. 10, 2–10 10.1046/j.1528-1157.44.s.1.7.x 14511388

[B15] Cardinali L. Jacobs S. Brozzoli C. Frassinetti F. Roy A. C. Farne A. (2012). Grab an object with a tool and change your body: tool-use-dependent changes of body representation for action. Exp. Brain Res. 218, 259–271 10.1007/s00221-012-3028-5 22349501

[B16] Cassidy J. D. Carroll L. J. Peloso P. M. Borg J. Von Holst H. Holm L. (2004). Incidence, risk factors and prevention of mild traumatic brain injury: results of the WHO collaborating centre task force on mild traumatic brain injury. J. Rehabil. Med. 43 Suppl, 28–60 15083870 10.1080/16501960410023732

[B17] Cernich A. N. Kurtz S. M. Mordecai K. L. Ryan P. B. (2010). Cognitive rehabilitation in traumatic brain injury. Curr. Treat. Options Neurol. 12, 412–423 10.1007/s11940-010-0085-6 20842598

[B18] Chen A. Y. Zagorski B. Parsons D. Van der Laan R. Chan V. Colantonio A. (2012). Factors associated with discharge destination from acute care after acquired brain injury in Ontario, Canada. BMC Neurol. 12:16 10.1186/1471-2377-12-16 22443681 PMC3402989

[B19] Cheng J. P. Shaw K. E. Monaco C. M. Hoffman A. N. Sozda C. N. Olsen A. S. (2012). A relatively brief exposure to environmental enrichment after experimental traumatic brain injury confers long-term cognitive benefits. J. Neurotrauma 29, 2684–2688 10.1089/neu.2012.2560 22774771 PMC3510443

[B20] Christensen B. K. Colella B. Inness E. Hebert D. Monette G. Bayley M. (2008). Recovery of cognitive function after traumatic brain injury: a multilevel modeling analysis of Canadian outcomes. Arch. Phys. Med. Rehabil. 89, S3–S15 10.1016/j.apmr.2008.10.002 19081439

[B21] Christensen H. Mackinnon A. (1993). The association between mental, social and physical activity and cognitive performance in young and old subjects. Age Ageing 22, 175–183 10.1093/ageing/22.3.175 8503313

[B22] Christodoulou C. Deluca J. Ricker J. H. Madigan N. K. Bly B. M. Lange G. (2001). Functional magnetic resonance imaging of working memory impairment after traumatic brain injury. J. Neurol. Neurosurg. Psychiatry 71, 161–168 10.1136/jnnp.71.2.161 11459886 PMC1737512

[B23] Churchill J. D. Galvez R. Colcombe S. Swain R. A. Kramer A. F. Greenough W. T. (2002). Exercise, experience and the aging brain. Neurobiol. Aging 23, 941–955 12392797 10.1016/s0197-4580(02)00028-3

[B24] Cicerone K. Mott T. Azulay J. Friel J. (2004). Community integration and satisfaction with functioning after intensive cognitive rehabilitation for traumatic brain injury. Arch. Phys. Med. Rehabil. 85, 943–950 10.1016/j.apmr.2003.07.019 15179648

[B25] Cicerone K. D. Dahlberg C. Kalmar K. Langenbahn D. M. Malec J. F. Bergquist T. F. (2000). Evidence-based cognitive rehabilitation: recommendations for clinical practice. Arch. Phys. Med. Rehabil. 81, 1596–1615 10.1053/apmr.2000.19240 11128897

[B26] Cicerone K. D. Dahlberg C. Malec J. F. Langenbahn D. M. Felicetti T. Kneipp S. (2005). Evidence-based cognitive rehabilitation: updated review of the literature from 1998 through 2002. Arch. Phys. Med. Rehabil. 86, 1681–19692 10.1016/j.apmr.2005.03.024 16084827

[B27] Cifu D. X. Kreutzer J. S. Kolakowsky-Hayner S. A. Marwitz J. H. Englander J. (2003). The relationship between therapy intensity and rehabilitative outcomes after traumatic brain injury: a multicenter analysis. Arch. Phys. Med. Rehabil. 84, 1441–1448 10.1016/S0003-9993(03)00272-7 14586910

[B28] C.I.H.I. (2006). Head injuries in Canada: a decade of change (1994-1995 to 2003-2004). National Trauma Regist. Anal. Brief. Available online at: http://secure.cihi.ca/cihiweb/dispPage.jsp?cw_page=bl_ntr_aug2006_e [Accessed February 10, 2010].

[B29] Cirillo J. Todd G. Semmler J. G. (2011). Corticomotor excitability and plasticity following complex visuomotor training in young and old adults. Eur. J. Neurosci. 34, 1847–1856 10.1111/j.1460-9568.2011.07870.x 22004476

[B30] Clarkson-Smith L. Hartley A. (1989). Relationships between physical exercise and cognitive abilities in older adults. Psychol. Aging 2, 183–189 2789745 10.1037//0882-7974.4.2.183

[B31] Corrigan J. D. Whiteneck G. Mellick D. (2004). Perceived needs following traumatic brain injury. J. Head Trauma Rehabil. 19, 205–216 15247843 10.1097/00001199-200405000-00002

[B32] Coulson E. J. Reid K. Shipham K. M. Morley S. Kilpatrick T. J. Bartlett P. F. (2004). The role of neurotransmission and the Chopper domain in p75 neurotrophin receptor death signaling. Prog. Brain Res. 146, 41–62 10.1016/S0079-6123(03)46003-2 14699955

[B33] Crowe M. Andel R. Pedersen N. Johansson B. Gatz M. (2003). Does participation in leisure activities lead to a reduced risk of Alzheimer's disease? A prospective study of Swedish twins. J. Gerontol. Ser. B 58B, 249–255 10.1093/geronb/58.5.P249 14507930

[B34] Davidson A. Bar-Yam Y. (2006). Environmental complexity: information for human-environment well-being in Unifying Themes in Complex Systems, eds Bar-Yam A. Minai Y. (New England; Berlin; Heidelberg: Springer), 157–168

[B35] De Weerdt W. Selz B. Nuyens G. Staes F. Swinnen D. Van De Winckel A. (2000). Time use of stroke patients in an intensive rehabilitation unit: a comparison between a Belgian and a Swiss setting. Disabil. Rehabil. 22, 181–186 10798306 10.1080/096382800296872

[B36] De Wit L. Putman K. Dejaeger E. Baert I. Berman P. Bogaerts K. (2005). Use of time by stroke patients: a comparison of four european rehabilitation centers. Stroke 36, 1977–1983 10.1161/01.STR.0000177871.59003.e3 16081860

[B37] De Witt B. W. Ehrenberg K. M. McAloon R. L. Panos A. H. Shaw K. E. Raghavan P. V. (2011). Abbreviated environmental enrichment enhances neurobehavioral recovery comparably to continuous exposure after traumatic brain injury. Neurorehabil. Neural Repair 25, 343–350 10.1177/1545968310390520 21186330 PMC3069145

[B38] Diamond M. (2001). Response of the brain to enrichment. An. Acad. Bras. Cienc. 73, 211–220 10.1590/S0001-37652001000200006 11404783

[B39] Dikmen S. Reitan R. M. Temkin N. R. (1983). Neuropsychological recovery in head injury. Arch. Neurol. 40, 333–338 10.1001/archneur.1983.04050060033004 6847436

[B40] Dikmen S. S. Ross B. L. Machamer J. E. Temkin N. R. (1995). One year psychosocial outcome in head injury. J. Int. Neuropsychol. Soc. 1, 67–77 9375211 10.1017/s1355617700000126

[B41] Dobrossy M. D. Dunnett S. B. (2001). The influence of environment and experience on neural grafts. Nat. Rev. Neurosci. 2, 871–879 10.1038/35104055 11733794

[B42] Dobrossy M. D. Dunnett S. B. (2004). Environmental enrichment affects striatal graft morphology and functional recovery. Eur. J. Neurosci. 19, 159–168 10.1111/j.1460-9568.2004.03105.x 14750974

[B43] Eckert M. J. Abraham W. C. (2012). Effects of environmental enrichment exposure on synaptic transmission and plasticity in the hippocampus in Current Topics in Behavorial Neurosciences, eds Geyer M. A. Ellenbroek B. A. Marsden C. A. (Berlin; Heidelberg: Springer), 1–23 10.1007/7854_2012_215 22798066

[B44] Eriksson G. Kottorp A. Borg J. Tham K. (2009). Relationship between occupational gaps in everyday life, depressive mood and life satisfaction after acquired brain injury. J. Rehabil. Med. 41, 187–194 10.2340/16501977-0307 19229453

[B45] Farbota K. D. Bendlin B. B. Alexander A. L. Rowley H. A. Dempsey R. J. Johnson S. C. (2012). Longitudinal diffusion tensor imaging and neuropsychological correlates in traumatic brain injury patients. Front. Hum. Neurosci. 6:160 10.3389/fnhum.2012.00160 22723773 PMC3378081

[B46] Fares R. P. Belmeguenai A. Sanchez P. E. Kouchi H. Y. Bodennec J. Morales A. (2013). Standardized environmental enrichment supports enhanced brain plasticity in healthy rats and prevents cognitive impairment in epileptic rats. PLoS ONE 8:e53888 10.1371/journal.pone.0053888 23342033 PMC3544705

[B47] Farne A. Buxbaum L. J. Ferraro M. Frassinetti F. Whyte J. Veramonti T. (2004). Patterns of spontaneous recovery of neglect and associated disorders in acute right brain-damaged patients. J. Neurol. Neurosurg. Psychiatr. 75, 1401–1410 10.1136/jnnp.2002.003095 15377685 PMC1738754

[B48] Fasotti L. Kovacs F. Eling P. Brouwer W. (2000). Time pressure management as a compensatory strategy training after closed head injury. Neuropsychol. Rehabil. 10, 47–65

[B49] Faul M. Xu L. Wald M. M. Coronado V. G. (2010). Traumatic Brain Injury in the United States: Emergency Department Visits, Hospitalizations, and Deaths. Atlanta: Centers for Disease Control and Prevention, National Center for Injury Prevention and Control

[B50] Fratiglioni L. Wang H. Ericsson K. Maytan M. Winblad B. (2000). Influence of social network on occurrence of dementia: a community-based longitudinal study. Lancet 355, 1315–1319 10.1016/S0140-6736(00)02113-9 10776744

[B51] Freeman E. A. (1997). Community-based rehabilitation of the person with a severe brain injury. Brain Inj. 11, 143–153 9012948 10.1080/026990597123746

[B52] Fujiwara Y. Chaves P. H. Yoshida H. Amano H. Fukaya T. Watanabe N. (2009). Intellectual activity and likelihood of subsequently improving or maintaining instrumental activities of daily living functioning in community-dwelling older Japanese: a longitudinal study. Int. J. Geriatr. Psychiatry 24, 547–555 10.1002/gps.2148 19132642

[B53] Gabriel A. Paoletti G. Della Seta D. Panelli R. Marcus M. Farabollini F. (2009a). Enriched environment and the recovery from inflammatory pain: social versus physical aspects and their interaction. Behav. Brain Res. 208, 90–95 10.1016/j.bbr.2009.11.015 19914294

[B54] Gabriel A. F. Marcus M. A. Honig W. M. Helgers N. Joosten E. A. (2009b). Environmental housing affects the duration of mechanical allodynia and the spinal astroglial activation in a rat model of chronic inflammatory pain. Brain Res. 1276, 83–90 10.1016/j.brainres.2009.04.039 19406110

[B55] Gan C. Gargaro J. Brandys C. Gerber G. Boschen K. (2010). Family caregivers' support needs after brain injury: a synthesis of perspectives from caregivers, programs, and researchers. NeuroRehabilitation 27, 5–18 10.3233/NRE-2010-0577 20634597

[B56] Gobbo O. L. O'Mara S. M. (2004). Impact of enriched-environment housing on brain-derived neurotrophic factor and on cognitive performance after a transient global ischemia. Behav. Brain Res. 152, 231–241 10.1016/j.bbr.2003.10.017 15196790

[B57] Green R. E. Colella B. Christensen B. Johns K. Frasca D. Bayley M. (2008). Examining moderators of cognitive recovery trajectories after moderate to severe traumatic brain injury. Arch. Phys. Med. Rehabil. 89, S16–S24 10.1016/j.apmr.2008.09.551 19081437

[B58] Green R. E. Melo B. Christensen B. Ngo L. Skene C. (2006). Evidence of transient enhancement to cognitive functioning in healthy young adults through environmental enrichment: implications for rehabilitation after brain injury. Brain Cogn. 60, 201–203 16646120

[B59] Greenberg G. Mikulis D. J. Ng K. Desouza D. Green R. E. (2008). Use of diffusion tensor imaging to examine subacute white matter injury progression in moderate to severe traumatic brain injury. Arch. Phys. Med. Rehabil. 89, S45–S50 10.1016/j.apmr.2008.08.211 19081441

[B60] Gribbin K. Schaie K. Parham I. (1980). Complexity of life style and maintenance of intellectual abilities. J. Soc. Issues 36, 47–61

[B61] Gunther V. K. Schafer P. Holzner B. J. Kemmler G. W. (2003). Long-term improvements in cognitive performance through computer-assisted cognitive training: a pilot study in a residential home for older people. Aging Ment. Health 7, 200–206 10.1080/1360786031000101175 12775401

[B62] Hamm R. Temple M. O'Dell D. Pike B. Lyeth B. (1996). Exposure to environmental complexity promotes recovery of cognitive function after traumtic brain injury. J. Neurotrauma 13, 41–47 8714862 10.1089/neu.1996.13.41

[B63] Hanks R. A. Rapport L. J. Wertheimer J. Koviak C. (2012). Randomized controlled trial of peer mentoring for individuals with traumatic brain injury and their significant others. Arch. Phys. Med. Rehabil. 93, 1297–1304 10.1016/j.apmr.2012.04.027 22840826

[B64] Hawthorne G. Gruen R. L. Kaye A. H. (2009). Traumatic brain injury and long-term quality of life: findings from an Australian study. J. Neurotrauma 26, 1623–1633 10.1089/neu.2008-0735 19317590

[B65] Hayden M. E. Plenger P. Bison K. Kowalske K. Masel B. Qualls D. (2013). Treatment effect versus pretreatment recovery in persons with traumatic brain injury: a study regarding the effectiveness of postacute rehabilitation. PM R. [Epub ahead of print]. 10.1016/j.pmrj.2012.12.005 23375632

[B66] Hayslip B. Maloy R. M. Kohl R. (1995). Long-term efficacy of fluid ability interventions with older adults. J. Gerontol. Ser. B Psychol. Sci. Soc. Sci. 50, P141–P149 7767692 10.1093/geronb/50b.3.p141

[B67] Hebb D. O. (1947). The effects of early experience on problem solving in maturity. Am. Psychol. 2, 306–307

[B68] Heffernan D. S. Vera R. M. Monaghan S. F. Thakkar R. K. Kozloff M. S. Connolly M. D. (2011). Impact of socioethnic factors on outcomes following traumatic brain injury. J. Trauma 70, 527–534 10.1097/TA.0b013e31820d0ed7 21610339

[B69] Heinemann A. W. Hamilton B. Linacre J. M. Wright B. D. Granger C. (1995). Functional status and therapeutic intensity during inpatient rehabilitation. Am. J. Phys. Med. Rehabil. 74, 315–326 7632391 10.1097/00002060-199507000-00011

[B70] Himanen L. Portin R. Isoniemi H. Helenius H. Kurki T. Tenovuo O. (2006). Longitudinal cognitive changes in traumatic brain injury: a 30-year follow-up study. Neurology 66, 187–192 10.1212/01.wnl.0000194264.60150.d3 16434651

[B71] Hoffman A. Malena R. Westergoma B. Luthra P. Chenga J. Aslama H. (2008). Environmental enrichment-mediated functional improvement after experimental traumatic brain injury is contingent on task-specific neurobehavioral experience. Neurosci. Lett. 431, 226–230 10.1016/j.neulet.2007.11.042 18162321 PMC3055238

[B72] Holbrook T. L. Anderson J. P. Sieber W. J. Browner D. Hoyt D. B. (1999). Outcome after major trauma: 12-month and 18-month follow-up results from the Trauma Recovery Project. J. Trauma 46, 765–771 discussion: 771–763. 10338392 10.1097/00005373-199905000-00003

[B73] Hoogerdijk B. Runge U. Haugboelle J. (2011). The adaptation process after traumatic brain injury an individual and ongoing occupational struggle to gain a new identity. Scand. J. Occup. Ther. 18, 122–132 10.3109/11038121003645985 20384550

[B74] Hudak A. Warner M. Marquez De La Plata C. Moore C. Harper C. Diaz-Arrastia R. (2011). Brain morphometry changes and depressive symptoms after traumatic brain injury. Psychiatry Res. 191, 160–165 10.1016/j.pscychresns.2010.10.003 21310594 PMC3053081

[B75] Huppert F. (1991). Age-Related Changes in Memory: Learning and Remembering New Information. Amsterdam: Elsevier Science Publishers

[B76] Johansson B. B. (2000). Brain plasticity and stroke rehabilitation: the Willis lecture. Stroke 31, 223–230 10.1161/01.STR.31.1.223 10625741

[B77] Johansson B. B. (2002). Environmental effects on recovery after stroke in Cerebrovascular Disease, 22nd Princeton Conference, ed Chan P. H. (New York, NY: Cambridge University Press), 328–336

[B78] Johansson B. B. (2003). Environmental influence on recovery after brain lesions - experimental and clinical data. J. Rehabil. Med. 35, 11–16 12817651 10.1080/16501960310010089

[B79] Johansson B. B. Ohlsson A. L. (1996). Environment, social interaction, and physical activity as determinants of functional outcome after cerebral infarction in the rat. Exp. Neurol. 139, 322–327 10.1006/exnr.1996.0106 8654535

[B80] Jung C. K. Herms J. (2012). Structural dynamics of dendritic spines are influenced by an environmental enrichment: an *in vivo* imaging study. Cereb. Cortex. [Epub ahead of print]. 10.1093/cercor/bhs317 23081882

[B81] Keane R. W. Kraydieh S. Lotocki G. Alonso O. F. Aldana P. Dietrich W. D. (2001). Apoptotic and antiapoptotic mechanisms after traumatic brain injury. J. Cereb. Blood Flow Metab. 21, 1189–1198 10.1097/00004647-200110000-00007 11598496

[B82] Keightley M. Kendall V. Jang S. H. Parker C. Agnihotri S. Colantonio A. (2011). From health care to home community: an Aboriginal community-based ABI transition strategy. Brain Inj. 25, 142–152 10.3109/02699052.2010.541898 21219087

[B83] Kempermann M. Gast G. Gage F. (2002). Neuroplasticity in old age: sustained fivefold induction of hippocampal neurogensis by long-term environmental enrichment. Ann. Neurol. 52, 135–143 10.1002/ana.10262 12210782

[B84] Kennedy M. Coelho C. Turkstra L. Ylvisaker M. Sohlberg M. Yorkston K. (2008). Intervention for executive functions after traumatic brain injury: a systematic review, meta-analysis and clinical recommendations. Neuropsychol. Rehabil. 18, 257–299 10.1080/09602010701748644 18569745

[B85] Kim J. Whyte J. Patel S. Avants B. Europa E. Wang J. (2010). Resting cerebral blood flow alterations in chronic traumatic brain injury: an arterial spin labeling perfusion FMRI study. J. Neurotrauma 27, 1399–1411 10.1089/neu.2009.1215 20528163 PMC2967826

[B86] Kleim J. A. Jones T. A. (2008). Principles of experience-dependent neural plasticity: implications for rehabilitation after brain damage. J. Speech Lang. Hear. Res. 51, S225–S239 10.1044/1092-4388(2008/018) 18230848

[B87] Klinge C. Roder B. Buchel C. (2012). Does training or deprivation modulate amygdala activation? Neuroimage 59, 1765–1771 10.1016/j.neuroimage.2011.08.046 21889991

[B88] Kobayashi S. Ohashi Y. Ando S. (2002). Effects of enriched environments with different durations and starting times on learning capacity during aging in rats assessed by a refined procedure of the Hebb-Williams maze task. J. Neurosci. Res. 70, 340–346 10.1002/jnr.10442 12391594

[B89] Kolb B. Whishaw I. (1998). Brain plasticity and behaviour. Annu. Rev. Psychol. 49, 43–64 10.1146/annurev.psych.49.1.43 9496621

[B90] Kramer A. F. Bherer L. Colcombe S. J. Dong W. Greenough W. T. (2004). Environmental influences on cognitive and brain plasticity during aging. J. Gerontol. Ser. A Biol. Sci. Med. Sci. 59, M940–M957 10.1093/gerona/59.9.M940 15472160

[B91] Latham N. Mason G. (2010). Frustration and perseveration in stereotypic captive animals: is a taste of enrichment worse than none at all? Behav. Brain Res. 211, 96–104 10.1016/j.bbr.2010.03.018 20230861

[B92] Leger M. Quiedeville A. Paizanis E. Natkunarajah S. Freret T. Boulouard M. (2012). Environmental enrichment enhances episodic-like memory in association with a modified neuronal activation profile in adult mice. PLoS ONE 7:e48043 10.1371/journal.pone.0048043 23110171 PMC3478271

[B93] Leon-Carrion J. Dominguez-Morales M. R. Barroso Y Martin J. M. Leon-Dominguez U. (2012). Recovery of cognitive function during comprehensive rehabilitation after severe traumatic brain injury. J. Rehabil. Med. 44, 505–511 10.2340/16501977-0982 22661001

[B94] Levine B. Kovacevic N. Nica E. I. Cheung G. Gao F. Schwartz M. L. (2008). The Toronto traumatic brain injury study: injury severity and quantified MRI. Neurology 70, 771–778 10.1212/01.wnl.0000304108.32283.aa 18316688

[B95] Lezak M. (2004). The neuropsycholgical examination: procedures in Neuropsychological Assessment, ed Lezak M. (New York, NY: Oxford University Press), 110–143

[B96] Lippert-Gruener M. Maegele M. Garbe J. Angelov D. N. (2007). Late effects of enriched environment (EE) plus multimodal early onset stimulation (MEOS) after traumatic brain injury in rats: ongoing improvement of neuromotor function despite sustained volume of the CNS lesion. Exp. Neurol. 203, 82–94 10.1016/j.expneurol.2006.07.025 16965773

[B97] Lores-Arnaiz S. Lores Arnaiz M. R. Czerniczyniec A. Cuello M. Bustamante J. (2010). Mitochondrial function and nitric oxide production in hippocampus and cerebral cortex of rats exposed to enriched environment. Brain Res. 1319, 44–53 10.1016/j.brainres.2010.01.017 20079718

[B98] Lundqvist A. Samuelsson K. (2012). Return to work after acquired brain injury: a patient perspective. Brain Inj. 26, 13–14 10.3109/02699052.2012.698363 22759203

[B99] Mackenzie J. D. Siddiqi F. Babb J. S. Bagley L. J. Mannon L. J. Sinson G. P. (2002). Brain atrophy in mild or moderate traumatic brain injury: a longitudinal quantitative analysis. AJNR Am. J. Neuroradiol. 23, 1509–1515 12372740 PMC7976797

[B100] Mackinnor A. Christensen H. Hofer S. Korten A. Jorm A. (2003). Use it and still lose it? The association between activity and cognitive performance established using latent growth techniques in a community sample. Aging Neuropsychol. Cogn. 10, 215–229

[B101] Mahncke H. Connor B. Appelman J. Ahsanuddin O. Hardy J. Wood R. (2006a). Memory enhancement in healthy older adults using a brain plasticity-based training program: a randomized, controlled study. Proc. Natl. Acad. Sci. U.S.A. 103, 12523–12528 10.1073/pnas.0605194103 16888038 PMC1526649

[B102] Mahncke H. W. Bronstone A. Merzenich M. M. (2006b). Brain plasticity and functional losses in the aged: scientific bases for a novel intervention. Prog. Brain Res. 157, 81–109 10.1016/S0079-6123(06)57006-2 17046669

[B103] Mahoney J. Eisner J. Havighurst T. Gray S. Palta M. (2000). Problems of older adults living alone after hospitalization. J. Gen. Int. Med. 15, 611–619 11029674 10.1046/j.1525-1497.2000.06139.x PMC1495595

[B104] Marioni R. E. Valenzuela M. J. Van Den Hout A. Brayne C. Matthews F. E. (2012a). Active cognitive lifestyle is associated with positive cognitive health transitions and compression of morbidity from age sixty-five. PLoS ONE 7:e50940 10.1371/journal.pone.0050940 23251404 PMC3521012

[B105] Marioni R. E. Van Den Hout A. Valenzuela M. J. Brayne C. Matthews F. E. (2012b). Active cognitive lifestyle associates with cognitive recovery and a reduced risk of cognitive decline. J. Alzheimers Dis. 28, 223–230 10.3233/JAD-2011-110377 21971400

[B106] Matter A. M. Folweiler K. A. Curatolo L. M. Kline A. E. (2010). Temporal effects of environmental enrichment-mediated functional improvement after experimental traumatic brain injury in rats. Neurorehabil. Neural Repair 25, 558–564 10.1177/1545968310397206 21436387 PMC3120632

[B107] McCormack E. Liddiard H. (2009). Home or away? Community rehabilitation following traumatic brain injury: a case report. Physiother. Res. Int. 14, 66–71 10.1002/pri.418 19107707

[B108] Meagher R. K. Mason G. J. (2012). Environmental enrichment reduces signs of boredom in caged mink. PLoS ONE 7:e49180 10.1371/journal.pone.0049180 23155462 PMC3498363

[B109] Milgram N. (2003). Cognitive experience and its effect on age-dependent cognitive decline in beagle dogs. Neurochem. Res. 2, 1677–1682 14584821 10.1023/a:1026009005108

[B110] Miller L. Green R. E. (in press). Can environmental enrichment serve as a protective intervention for neurodegeneration in traumatic brain injury? Front. Hum. Neurosci.

[B111] Millis S. R. Rosenthal M. Novack T. A. Sherer M. Nick T. G. Kreutzer J. S. (2001). Long-term neuropsychological outcome after traumatic brain injury. J. Head Trauma Rehabil. 16, 343–355 11461657 10.1097/00001199-200108000-00005

[B112] Mohammed A. K. Winblad B. Ebendal T. Larkfors L. (1990). Environmental influence on behaviour and nerve growth factor in the brain. Brain Res. 528, 62–72 10.1016/0006-8993(90)90195-H 2245339

[B113] Monaco C. M. Mattiola V. V. Folweiler K. A. Tay J. K. Yelleswarapu N. K. Curatolo L. M. (2013). Environmental enrichment promotes robust functional and histological benefits in female rats after controlled cortical impact injury. Exp. Neurol. [Epub ahead of print]. 10.1016/j.expneurol.2013.01.007 23333563 PMC3644389

[B114] Murray C. J. Lopez A. D. (1997). Alternative projections of mortality and disability by cause 1990-2020: Global Burden of Disease Study. Lancet 349, 1498–1504 10.1016/S0140-6736(96)07492-2 9167458

[B115] Nalder E. Fleming J. Cornwell P. Foster M. Haines T. (2012a). Factors associated with the occurrence of sentinel events during transition from hospital to home for individuals with traumatic brain injury. J. Rehabil. Med. 44, 837–844 10.2340/16501977-1033 22960731

[B116] Nalder E. Fleming J. Foster M. Cornwell P. Shields C. Khan A. (2012b). Identifying factors associated with perceived success in the transition from hospital to home after brain injury. J. Head Trauma Rehabil. 27, 143–153 10.1097/HTR.0b013e3182168fb1 21522027

[B117] Narayan R. K. Michel M. E. Ansell B. Baethmann A. Biegon A. Bracken M. B. (2002). Clinical trials in head injury. J. Neurotrauma 19, 503–557 10.1089/089771502753754037 12042091 PMC1462953

[B118] Neely A. S. Backman L. (1995). Effects of multifactorial memory training in old age: generalizability across tasks and individuals. J. Gerontol. Ser. B Psychol. Sci. Soc. Sci. 50, P134–P140 7767691 10.1093/geronb/50b.3.p134

[B119] Newson R. Kemps E. (2005). General lifestyle activities as a predictor of current cognition and cognitive change in older adults: a cross-sectional and longitudinal examination. J. Gerontol. 60B, P113 10.1093/geronb/60.3.P113 15860780

[B120] Ng K. Mikulis D. J. Glazer J. Kabani N. Till C. Greenberg G. (2008). Magnetic resonance imaging evidence of progression of subacute brain atrophy in moderate to severe traumatic brain injury. Arch. Phys. Med. Rehabil. 89, S35–S44 10.1016/j.apmr.2008.07.006 19081440

[B121] Nilsson M. Perfilieva E. Johansson U. Orwar O. Eriksson P. S. (1999). Enriched environment increases neurogenesis in the adult rat dentate gyrus and improves spatial memory. J. Neurobiol. 39, 569–578 10.1002/(SICI)1097-4695(19990615)39:4<569::AID-NEU10>3.0.CO;2-F 10380078

[B122] Noice H. Noice T. (2004). A short term intervention to enhance cognitive and affective functioning in older adults. J. Aging Health 16, 562–585 10.1177/0898264304265819 15271270

[B123] Paillard-Borg S. Fratiglioni L. Xu W. Winblad B. Wang H. X. (2012). An active lifestyle postpones dementia onset by more than one year in very old adults. J. Alzheimers Dis. 31, 835–842 10.3233/JAD-2012-120724 22751170

[B124] Park N. W. Ingles J. L. (2001). Effectiveness of attention rehabilitation after an acquired brain injury: a meta-analysis. Neuropsychology 15, 199–210 11324863 10.1037//0894-4105.15.2.199

[B125] Passineau M. Green E. Dalton D. (2001). Therapeutic effects of environmental enrichment on cognitive function and tissue integrity following severe traumatic brain injury in rats. Exp. Neurol. 168, 373–384 10.1006/exnr.2000.7623 11259125

[B126] Pereira L. O. Arteni N. S. Petersen R. C. Da Rocha A. P. Achaval M. Netto C. A. (2007). Effects of daily environmental enrichment on memory deficits and brain injury following neonatal hypoxia-ischemia in the rat. Neurobiol. Learn. Mem. 87, 101–108 10.1016/j.nlm.2006.07.003 16931063

[B127] Pickett W. Simpson K. Brison R. J. (2004). Rates and external causes of blunt head trauma in Ontario: analysis and review of Ontario Trauma Registry datasets. Chronic Dis. Can. 25, 32–41 15298486

[B128] Pietropaolo S. Branchi I. Cirulli F. Chiarotti F. Aloe L. Alleva E. (2004). Long-term effects of the periadolescent environment on exploratory activity and aggressive behaviour in mice: social versus physical enrichment. Physiol. Behav. 81, 443–453 10.1016/j.physbeh.2004.02.022 15135016

[B129] Pignatti F. Rozzini R. Trabucchi M. (2002). Physical activity and cognitive decline in elderly persons. Arch. Intern. Med. 162, P361 11822936 10.1001/archinte.162.3.361

[B130] Powell J. Heslin J. Greenwood R. (2002). Community based rehabilitation after severe traumatic brain injury: a randomised controlled trial. J. Neurol. Neurosurg. Psychiatr. 72, 193–202 10.1136/jnnp.72.2.193 11796769 PMC1737759

[B131] Pressman H. T. (2007). Traumatic brain injury rehabilitation: case management and insurance-related issues. Phys. Med. Rehabil. Clin. N. Am. 18, 165–174 viii. 10.1016/j.pmr.2006.11.006 17292818

[B132] Pushkar D. Arbuckle T. Conway M. Chaikelson J. Maag U. (1997). Everyday activity parameters and competence in older adults. Psychol. Aging 12, 600–609 9416629 10.1037//0882-7974.12.4.600

[B133] Qiu X. Huang C. X. Lu W. Yang S. Li C. Shi X. Y. (2012). Effects of a 4 month enriched environment on the hippocampus and the myelinated fibers in the hippocampus of middle-aged rats. Brain Res. 1465, 26–33 10.1016/j.brainres.2012.05.025 22627162

[B134] Qiu X. Li C. Jiang R. Chen L. Huang C. Yang S. (2011). The effects of short-term enriched environment on capillaries of the middle-aged rat cortex. Neurosci. Lett. 505, 186–190 10.1016/j.neulet.2011.10.019 22019983

[B135] Raghupathi R. (2004). Cell death mechanisms following traumatic brain injury. Brain Pathol. 14, 215–222 15193035 10.1111/j.1750-3639.2004.tb00056.x PMC8096005

[B136] Raghupathi R. Graham D. I. McIntosh T. K. (2000). Apoptosis after traumatic brain injury. J. Neurotrauma 17, 927–938 11063058 10.1089/neu.2000.17.927

[B137] Rath J. Simon D. Langenbahn D. Sherr R. Diller L. (2003). Group treatment of problem-solving deficits in outpatients with traumatic brain injury: a randomised outcome study. Neuropsychol. Rehabil. 13, 461–488

[B138] Resch J. A. Villarreal V. Johnson C. L. Elliott T. R. Kwok O. M. Berry J. W. (2009). Trajectories of life satisfaction in the first 5 years following traumatic brain injury. Rehabil. Psychol. 54, 51–59 19618703 10.1037/a0015051

[B139] Rittman M. Faircloth C. Boylstein C. Gubrium J. F. Williams C. Van Puymbroeck M. (2004). The experience of time in the transition from hospital to home following stroke. J. Rehabil. Res. Dev. 41, 259–268 15543443 10.1682/jrrd.2003.06.0099

[B140] Rosenzweig M. (1966). Environmental complexity, cerebral change, and behavior. Am. Psychol. 21, 321–332 5910063 10.1037/h0023555

[B141] Rosenzweig M. Bennett E. (1996). Psychobiology of plasticity: effects of training and experience on brain and behaviour. Behav. Brain Res. 78, 57–65 10.1016/0166-4328(95)00216-2 8793038

[B142] Rotondi A. J. Sinkule J. Balzer K. Harris J. Moldovan R. (2007). A qualitative needs assessment of persons who have experienced traumatic brain injury and their primary family caregivers. J. Head Trauma Rehabil. 22, 14–25 17235227 10.1097/00001199-200701000-00002

[B143] Rubinov M. McIntosh A. R. Valenzuela M. J. Breakspear M. (2009). Simulation of neuronal death and network recovery in a computational model of distributed cortical activity. Am. J. Geriatr. Psychiatry 17, 210–217 10.1097/JGP.0b013e318187137a 19001355

[B144] Ruff R. Barth J. Kreutzer J. Levin H. Foulkes M. (1991). Verbal learning deficits following severe head injury: heterogeneity in recovery over 1 year. J. Neurosurg. 75, S50–S58

[B145] Rumrill P. D. Fitzgerald S. M. Merchant W. R. (2010). Using scoping literature reviews as a means of understanding and interpreting existing literature. Work 35, 399–404 10.3233/WOR-2010-0998 20364059

[B146] Rusconi S. Turner-Stokes L. (2003). An evaluation of aftercare following discharge from a specialist in-patient rehabilitation service. Disabil. Rehabil. 25, 1281–1288 10.1080/09638280310001608582 14617446

[B147] Salmond C. H. Menon D. K. Chatfield D. A. Pickard J. D. Sahakian B. J. (2006). Changes over time in cognitive and structural profiles of head injury survivors. Neuropsychologia 44, 1995–1998 10.1016/j.neuropsychologia.2006.03.013 16620889

[B148] Salthouse T. (2006). Mental exercise and mental aging: evaluating the validity of the “use it or lose it” hypothesis. Perspect. Psychol. Sci. 1, 68–87 10.1111/j.1745-6916.2006.00005.x 26151186

[B149] Sampson E. L. Bulpitt C. J. Fletcher A. E. (2009). Survival of community-dwelling older people: the effect of cognitive impairment and social engagement. J. Am. Geriatr. Soc. 57, 985–991 10.1111/j.1532-5415.2009.02265.x 19507292

[B150] Sander A. M. Clark A. Pappadis M. R. (2010). What is community integration anyway?: defining meaning following traumatic brain injury. J. Head Trauma Rehabil. 25, 121–127 10.1097/HTR.0b013e3181cd1635 20134333

[B151] Sander A. M. Pappadis M. R. Clark A. N. Struchen M. A. (2011). Perceptions of community integration in an ethnically diverse sample. J. Head Trauma Rehabil. 26, 158–169 10.1097/HTR.0b013e3181e7537e 20631629

[B152] Sander A. M. Pappadis M. R. Davis L. C. Clark A. N. Evans G. Struchen M. A. (2009). Relationship of race/ethnicity and income to community integration following traumatic brain injury: investigation in a non-rehabilitation trauma sample. NeuroRehabilitation 24, 15–27 10.3233/NRE-2009-0450 19208954

[B153] Sander A. M. Roebuck T. M. Struchen M. A. Sherer M. High W. M. (2001). Long-term maintenance of gains obtained in postacute rehabilitation by persons with traumatic brain injury. J. Head Trauma Rehabil. 16, 356–373 11461658 10.1097/00001199-200108000-00006

[B154] Scarmeas N. Stern Y. (2003). Cognitive reserve and lifestyle. J. Clin. Exp. Neuropsychol. 25, 625–633 10.1076/jcen.25.5.625.14576 12815500 PMC3024591

[B155] Schooler C. (1987). Psychological effects of complex environments during the life span: a review and theory in Cognitive Functioning and Social Structure Over the Life Course, ed Schaie C. S. K. W. (Norwood, NJ: Ablex), 24–49

[B156] Schooler C. Mulatu M. (2001). The reciprocal effects of leisure time activities and intellectual functioning in older people: a longitudinal analysis. Psychol. Aging 16, 466–482 11554524 10.1037//0882-7974.16.3.466

[B157] Schuit A. Feskens E. Launer L. Kromhout D. (2001). Physical activity and cognitive decline, the role of apolipoprotein e4 allele. Med. Sci. Sports Exerc. 33, 772–777 11323547 10.1097/00005768-200105000-00015

[B158] Seeman T. Lusignolo T. Albert M. Berkman L. (2001). Social relationships, social support, and patterns of cognitive aging in healthy, high-functioning older adults: MacArthur studies of successful aging. Health Psychol. 20, 243–255 11515736 10.1037//0278-6133.20.4.243

[B159] Shafi S. Marquez De La Plata C. Diaz-Arrastia R. Shipman K. Carlile M. Frankel H. (2007). Racial disparities in long-term functional outcome after traumatic brain injury. J. Trauma 63, 1263–1268 discussion: 1268–1270. 10.1097/TA.0b013e31815b8f00 18212648

[B160] Shiel A. Burn J. P. Henry D. Clark J. Wilson B. A. Burnett M. E. (2001). The effects of increased rehabilitation therapy after brain injury: results of a prospective controlled trial. Clin. Rehabil. 15, 501–514 10.1191/026921501680425225 11594640

[B161] Shigaki C. L. Johnstone B. Schopp L. H. (2009). Financial and vocational outcomes 2 years after traumatic brain injury. Disabil. Rehabil. 31, 484–489 10.1080/09638280802240449 19034724

[B162] Shin S. S. Bales J. W. Yan H. Q. Kline A. E. Wagner A. K. Lyons-Weiler J. (2013). The effect of environmental enrichment on substantia nigra gene expression after traumatic brain injury in rats. J. Neurotrauma 30, 259–270 10.1089/neu.2012.2462 23094804 PMC3579325

[B163] Shors T. J. Anderson M. L. Curlik D. M. 2nd. Nokia M. S. (2012). Use it or lose it: how neurogenesis keeps the brain fit for learning. Behav. Brain Res. 227, 450–458 10.1016/j.bbr.2011.04.023 21536076 PMC3191246

[B164] Sidaros A. Engberg A. W. Sidaros K. Liptrot M. G. Herning M. Petersen P. (2008). Diffusion tensor imaging during recovery from severe traumatic brain injury and relation to clinical outcome: a longitudinal study. Brain 131, 559–572 10.1093/brain/awm294 18083753

[B165] Sidaros A. Skimminge A. Liptrot M. G. Sidaros K. Engberg A. W. Herning M. (2009). Long-term global and regional brain volume changes following severe traumatic brain injury: a longitudinal study with clinical correlates. Neuroimage 44, 1–8 10.1016/j.neuroimage.2008.08.030 18804539

[B166] Simpson J. Kelly J. P. (2011). The impact of environmental enrichment in laboratory rats–behavioural and neurochemical aspects. Behav. Brain Res. 222, 246–264 10.1016/j.bbr.2011.04.002 21504762

[B167] Slade A. Tennant A. Chamberlain M. A. (2002). A randomised controlled trial to determine the effect of intensity of therapy upon length of stay in a neurological rehabilitation setting. J. Rehabil. Med. 34, 260–266 12440799 10.1080/165019702760390347

[B168] SMARTRISK. (2006). The Economic Burden of Injury. Toronto, ON: SMARTRISK

[B169] Sohlberg M. M. McLaughlin K. A. Pavese A. Heidrich A. Posner M. I. (2000). Evaluation of attention process training and brain injury education in persons with acquired brain injury. J. Clin. Exp. Neuropsychol. 22, 656–676 10.1076/1380-3395(200010)22:5;1-9;FT656 11094401

[B170] Sozda C. N. Hoffman A. N. Olsen A. S. Cheng J. P. Zafonte R. D. Kline A. E. (2010). Empirical comparison of typical and atypical environmental enrichment paradigms on functional and histological outcome after experimental traumatic brain injury. J. Neurotrauma 27, 1047–1057 10.1089/neu.2010.1313 20334496 PMC2943502

[B171] Speisman R. B. Kumar A. Rani A. Pastoriza J. M. Severance J. E. Foster T. C. (2012). Environmental enrichment restores neurogenesis and rapid acquisition in aged rats. Neurobiol. Aging 34, 263–274 10.1016/j.neurobiolaging.2012.05.023 22795793 PMC3480541

[B172] Spikman J. M. Boelen D. H. Lamberts K. F. Brouwer W. H. Fasotti L. (2009). Effects of a multifaceted treatment program for executive dysfunction after acquired brain injury on indications of executive functioning in daily life. J. Int. Neuropsychol. Soc. 16, 118–129 10.1017/S1355617709991020 19900348

[B173] Spivack G. Spettell C. M. Ellis D. W. Ross S. E. (1992). Effects of intensity of treatment and length of stay on rehabilitation outcomes. Brain Inj. 6, 419–434 1393175 10.3109/02699059209008138

[B174] Staudenmayer K. L. Diaz-Arrastia R. De Oliveira A. Gentilello L. M. Shafi S. (2007). Ethnic disparities in long-term functional outcomes after traumatic brain injury. J. Trauma 63, 1364–1369 10.1097/TA.0b013e31815b897b 18212662

[B175] Stine-Morrow E. Parisi J. Morrow D. Greene J. Park D. (2007). An engagement model of cognitive optimization through adulthood. J. Gerontol. Ser. B 62B, 62–69 17565166 10.1093/geronb/62.special_issue_1.62

[B176] Strandberg T. (2009). Adults with acquired traumatic brain injury: experiences of a changeover process and consequences in everyday life. Soc. Work Health Care 48, 276–297 10.1080/00981380802599240 19360531

[B177] Struchen M. A. Davis L. C. Bogaards J. A. Hudler-Hull T. Clark A. N. Mazzei D. M. (2011). Making connections after brain injury: development and evaluation of a social peer-mentoring program for persons with traumatic brain injury. J. Head Trauma Rehabil. 26, 4–19 10.1097/HTR.0b013e3182048e98 21209559

[B178] Sun H. Zhang J. Zhang L. Liu H. Zhu H. Yang Y. (2010). Environmental enrichment influences BDNF and NR1 levels in the hippocampus and restores cognitive impairment in chronic cerebral hypoperfused rats. Curr. Neurovasc. Res. 7, 268–280 10.2174/156720210793180819 20854252

[B179] Till C. Colella B. Verwegen J. Green R. E. (2008). Postrecovery cognitive decline in adults with traumatic brain injury. Arch. Phys. Med. Rehabil. 89, S25–S34 10.1016/j.apmr.2008.07.004 19081438

[B180] Toglia J. Johnston M. V. Goverover Y. Dain B. (2010). A multicontext approach to promoting transfer of strategy use and self regulation after brain injury: an exploratory study. Brain Inj. 24, 664–677 10.3109/02699051003610474 20235769

[B181] Toglia J. P. (1991). Generalization of treatment: a multicontext approach to cognitive perceptual impairment in adults with brain injury. Am. J. Occup. Ther. 45, 505–516 10.5014/ajot.45.6.505 2063940

[B182] Trivedi M. A. Ward M. A. Hess T. M. Gale S. D. Dempsey R. J. Rowley H. A. (2007). Longitudinal changes in global brain volume between 79 and 409 days after traumatic brain injury: relationship with duration of coma. J. Neurotrauma 24, 766–771 10.1089/neu.2006.0205 17518532 PMC2627781

[B183] Tsaousides T. Warshowsky A. Ashman T. A. Cantor J. B. Spielman L. Gordon W. A. (2009). The relationship between employment-related self-efficacy and quality of life following traumatic brain injury. Rehabil. Psychol. 54, 299–305 10.1037/a0016807 19702428

[B184] Turner B. Fleming J. Cornwell P. Worrall L. Ownsworth T. Haines T. (2007). A qualitative study of the transition from hospital to home for individuals with acquired brain injury and their family caregivers. Brain Inj. 21, 1119–1130 10.1080/02699050701651678 17952712

[B185] Turner B. Fleming J. Cornwell P. Haines T. Ownsworth T. (2009a). Profiling early outcomes during the transition from hospital to home after brain injury. Brain Inj. 23, 51–60 10.1080/02699050802635257 19172450

[B186] Turner B. Ownsworth T. Cornwell P. Fleming J. (2009b). Reengagement in meaningful occupations during the transition from hospital to home for people with acquired brain injury and their family caregivers. Am. J. Occup. Ther. 63, 609–620 10.5014/ajot.63.5.609 19785260

[B187] Turner B. Fleming J. Ownsworth T. Cornwell P. (2011a). Perceptions of recovery during the early transition phase from hospital to home following acquired brain injury: a journey of discovery. Neuropsychol. Rehabil. 21, 64–91 10.1080/09602011.2010.527747 21132603

[B188] Turner B. J. Fleming J. Ownsworth T. Cornwell P. (2011b). Perceived service and support needs during transition from hospital to home following acquired brain injury. Disabil. Rehabil. 33, 818–829 10.3109/09638288.2010.513422 20812814

[B189] Turner B. J. Fleming J. M. Ownsworth T. L. Cornwell P. L. (2008). The transition from hospital to home for individuals with acquired brain injury: a literature review and research recommendations. Disabil. Rehabil. 30, 1153–1176 10.1080/09638280701532854 17852241

[B190] Turner G. R. Green E. (2008). Cognitive remediation in aging and ABI: a question of negative plasticity? Neuropsychol. Rehabil. 18, 372–384

[B191] Turner-Stokes L. Disler P. B. Nair A. Wade D. T. (2005). Multi-disciplinary rehabilitation for acquired brain injury in adults of working age. Cochrane Database Syst. Rev. 3:CD004170 10.1002/14651858.CD004170.pub2 16034923

[B192] Valero J. Espana J. Parra-Damas A. Martin E. Rodriguez-Alvarez J. Saura C. A. (2011). Short-term environmental enrichment rescues adult neurogenesis and memory deficits in APP(Sw, Ind) transgenic mice. PLoS ONE 6:e16832 10.1371/journal.pone.0016832 21347380 PMC3036721

[B193] Van De Winckel A. Feys H. De Weerdt W. Dom R. (2004). Cognitive and behavioural effects of music-based exercises in patients with dementia. Clin. Rehabil. 18, 253–260 10.1191/0269215504cr750oa 15137556

[B194] Van Praag H. Kempermann G. Gage F. (2000). Neural consequences of environmental enrichment. Neuroscience 1, 191–198 10.1038/35044558 11257907

[B195] Vance D. Johns R. (2002). Montessori improved cognitive domains in adults with Alzheimer's Disease. Phys. Occup. Ther. Geriatr. 20, 19–36

[B196] Vazquez-Sanroman D. Sanchis-Segura C. Toledo R. Hernandez M. E. Manzo J. Miquel M. (2013). The effects of enriched environment on BDNF expression in the mouse cerebellum depending on the length of exposure. Behav. Brain Res. 243C, 118–128 10.1016/j.bbr.2012.12.047 23295397

[B197] Verghese J. Lipton R. Katz M. Hall C. Derby C. Kuslansky G. (2003). Leisure activities and the risk of dementia in the elderly. N. Engl. J. Med. 348, 2508–2616 10.1056/NEJMoa022252 12815136

[B198] Voss M. W. Nagamatsu L. S. Liu-Ambrose T. Kramer A. F. (2011). Exercise, brain, and cognition across the life span. J. Appl. Physiol. 111, 1505–1513 10.1152/japplphysiol.00210.2011 21527670 PMC3220305

[B199] Wang H. Karp A. Wonblad B. Fratiglioni L. (2002). Late-life engagement in social and leisure activities is associated with a decreased risk of dementia: a longitudinal study from the Kungsholmen project. Am. J. Epidemiol. 155, 1081–1087 10.1093/aje/155.12.1081 12048221

[B200] Warner M. A. Marquez De La Plata C. Spence J. Wang J. Y. Harper C. Moore C. (2010). Assessing spatial relationships between axonal integrity, regional brain volumes, and neuropsychological outcomes after traumatic axonal injury. J. Neurotrauma 27, 2121–2130 10.1089/neu.2010.1429 20874032 PMC2996819

[B201] Warraich Z. Kleim J. A. (2010). Neural plasticity: the biological substrate for neurorehabilitation. PM R 2, S208–S219 10.1016/j.pmrj.2010.10.016 21172683

[B202] West R. W. Greenough W. T. (1972). Effect of environmental complexity on cortical synapses of rats: preliminary results. Behav. Biol. 7, 279–284 5041867 10.1016/s0091-6773(72)80207-4

[B203] Will B. Galani R. Kelche C. Rosenzweig M. R. (2004). Recovery from brain injury in animals: relative efficacy of environmental enrichment, physical exercise or formal training (1990-2002). Prog. Neurobiol. 72, 167–182 10.1016/j.pneurobio.2004.03.001 15130708

[B204] Willer B. Button J. Rempel R. (1999). Residential and home-based postacute rehabilitation of individuals with traumatic brain injury: a case control study. Arch. Phys. Med. Rehabil. 80, 399–406 10206601 10.1016/s0003-9993(99)90276-9

[B205] Williamson L. L. Chao A. Bilbo S. D. (2012). Environmental enrichment alters glial antigen expression and neuroimmune function in the adult rat hippocampus. Brain Behav. Immun. 26, 500–510 10.1016/j.bbi.2012.01.003 22281279 PMC3294275

[B206] Wilson J. T. Wiedmann K. D. Hadley D. M. Condon B. Teasdale G. Brooks D. N. (1988). Early and late magnetic resonance imaging and neuropsychological outcome after head injury. J. Neurol. Neurosurg. Psychiatr. 51, 391–396 10.1136/jnnp.51.3.391 3361330 PMC1032866

[B207] Wilson R. Barnes L. Bennett D. (2003). Assessment of lifetime participation in cognitively stimulating activities. J. Clin. Exp. Neuropsychol. 25, 634–642 10.1076/jcen.25.5.634.14572 12815501

[B208] Wilson R. S. Mendes De Leon C. F. Barnes L. L. Schneider J. A. Bienias J. L. Evans D. A. (2002). Participation in cognitively stimulating activities and risk of incident Alzheimer disease. J. Am. Med. Assoc. 287, 742–748 10.1001/jama.287.6.742 11851541

[B209] Winocur G. Moscovitch M. (1990). A comparison of cognitive function in community-dwelling and institutionalized old people of normal intelligence. Can. J. Psychol. 44, 435–444 2292085 10.1037/h0084270

[B210] Winocur G. Moscovitch M. Freedman J. (1987). An investigation of cognitive function in relation to psychosocial variables in institutionalized old people. Can. J. Psychol. 41, 257–269 3502900 10.1037/h0084156

[B211] Winocur G. (1998). Environmental influences on cognitive decline in aged rats. Neurobiol. Aging 19, 589–597 10192219 10.1016/s0197-4580(98)00107-9

[B212] Xu L. Jiang C. Q. Lam T. H. Zhang W. S. Thomas G. N. Cheng K. K. (2011). Dose-response relation between physical activity and cognitive function: guangzhou biobank cohort study. Ann. Epidemiol. 21, 857–863 10.1016/j.annepidem.2011.06.002 21784658

[B213] Yang S. Li C. Qiu X. Zhang L. Lu W. Chen L. (2013). Effects of an enriched environment on myelin sheaths in the white matter of rats during normal aging: a stereological study. Neuroscience 234C, 13–21 10.1016/j.neuroscience.2013.01.003 23313226

[B214] Zhu X. L. Poon W. S. Chan C. H. Chan S. H. (2001). Does intensive rehabilitation improve the functional outcome of patients with traumatic brain injury? Interim result of a randomized controlled trial. Br. J. Neurosurg. 15, 464–473 11813997 10.1080/02688690120097688

[B215] Zhu X. L. Poon W. S. Chan C. C. Chan S. S. (2007). Does intensive rehabilitation improve the functional outcome of patients with traumatic brain injury (TBI)? A randomized controlled trial. Brain Inj. 21, 681–690 10.1080/02699050701468941 17653942

